# Visuomotor anomalies in achiasmatic mice expressing a transfer-defective Vax1 mutant

**DOI:** 10.1038/s12276-023-00930-4

**Published:** 2023-02-03

**Authors:** Kwang Wook Min, Namsuk Kim, Jae Hoon Lee, Younghoon Sung, Museong Kim, Eun Jung Lee, Jong-Myeong Kim, Jae-Hyun Kim, Jaeyoung Lee, Wonjin Cho, Jee Myung Yang, Nury Kim, Jaehoon Kim, C. Justin Lee, Young-Gyun Park, Seung-Hee Lee, Han-Woong Lee, Jin Woo Kim

**Affiliations:** 1 Department of Biological Sciences and KAIST Stem Cell Center, Korea Advanced Institute of Science and Technology, Daejeon, 34141 Republic of Korea; 2 Neurovascular Unit, Korea Brain Research Institute, Daegu, 41062 Republic of Korea; 3 Department of Biochemistry, College of Life Science and Biotechnology, Yonsei University, Seoul, 03722 Republic of Korea; 4 Department of Convergence Medicine, University of Ulsan College of Medicine, Seoul, 05505 Republic of Korea; 5 Department of Bio & Brain Engineering, Korea Advanced Institute of Science and Technology, Daejeon, 34141 Republic of Korea; 6 Department of Ophthalmology, Dongguk University Ilsan Hospital, Dongguk-ro 27, Ilsandong-gu, Goyang, Gyeong-gi Republic of Korea; 7 Center for Cognition and Sociality, Institute for Basic Science, Daejeon, 34126 Republic of Korea

**Keywords:** Axon and dendritic guidance, Morphogen signalling

## Abstract

In binocular animals that exhibit stereoscopic visual responses, the axons of retinal ganglion cells (RGCs) connect to brain areas bilaterally by forming a commissure called the optic chiasm (OC). Ventral anterior homeobox 1 (Vax1) contributes to the formation of the OC, acting endogenously in optic pathway cells and exogenously in growing RGC axons. Here, we generated *Vax1*^*AA/AA*^ mice expressing the Vax1^AA^ mutant, which is incapable of intercellular transfer. We found that RGC axons cannot take up Vax1^AA^ protein from the *Vax1*^*AA/AA*^ mouse optic stalk (OS) and grow slowly to arrive at the hypothalamus at a late stage. The RGC axons of *Vax1*^*AA/AA*^ mice connect exclusively to ipsilateral brain areas after failing to access the midline, resulting in reduced visual acuity and abnormal oculomotor responses. Overall, our study provides physiological evidence for the necessity of intercellular transfer of Vax1 and the importance of the bilateral RGC axon projection in proper visuomotor responses.

## Vision: bringing the two sides together

A protein regulating gene expression called Vax1 is essential for building the optic chiasm (OC), a brain structure where nerves from the left and right eyes cross to the other side of the brain. Signals from both eyes must be integrated for space and depth perception. Although some cues guiding optic nerve growth are known, those for the OC are not. Jin Woo Kim at the Korea Advanced Institute of Science and Technology, Daejeon, South Korea, and co-workers investigated visual development in mice expressing a mutant Vax1. They found that the mutant Vax1 could not support the optic nerve, which grew slowly and failed to connect to the other side of the brain. Consequently, the mice had impaired depth perception and low vision. These results show the importance of Vax1 for the development of binocular vision.

## Introduction

Animals collect visual information through the eyes, where photoreceptors in the retina convert light stimuli into electrochemical signals^1^. The signals are then transmitted to inner retinal neural circuits before being sent to the brain via retinal ganglion cells (RGCs). RGC axons, which are bundled in the optic nerve, deliver visual information to multiple brain areas, including the dorsal lateral geniculate nucleus (dLGN) of the thalamus, for pattern and color recognition; the superior colliculus (SC) of the midbrain, for oculomotor responses; and the suprachiasmatic nucleus (SCN) of the hypothalamus, for circadian rhythm control^1–3^.

In many binocular animals, RGC axons are not only wired to brain areas on the same (i.e., ipsilateral) side, but are also connected to those on the opposite (i.e., contralateral) side^4,5^. The population of RGCs whose axons project to the ipsilateral brain areas is variable among vertebrate species. Human RGC axons are split equally to both sides at the midline, whereas all RGC axons extend exclusively to the contralateral side across the midline in *Xenopus laevis* and zebrafish^4,5^. In mice, only a minority of RGCs connect to the ipsilateral brain areas: ~3% in pigmented mice and ~1% in albino mice^6^.

RGC axons form a midline structure called the optic chiasm (OC), which is located beneath the SCN and splits the axon bundles into ipsilateral and contralateral paths^4,5^. Pathway selection for RGC axons at the OC is determined by specific guidance cues. For example, Ephrin-B2 and -B3 expressed in radial glia of the ventral hypothalamus (vHT) act through a receptor, EphB1, in RGC axons from the ventral and temporal (VT) retina to repel the axons from the midline, guiding their growth ipsilaterally^7^. EphB1 is present in approximately 50% of human RGCs and ~3% of mouse RGCs but is absent in *Xenopus* and zebrafish RGCs, suggesting a critical role of EphB1 in ipsilateral pathway selection by RGC axons^4,5^. Pathway-selection cues are provided not only by cells located along RGC axon growth tracks but also by neighboring RGC axons. Sonic hedgehog (Shh), which is expressed in contralaterally projecting RGC axons, also serves as a repulsive cue in the OC by acting on its coreceptor, Boc, which is expressed in RGC axons from the VT mouse retina^8^.

The cues that guide the majority of mouse RGC axons across the midline, however, have not been identified as clearly as the ipsilateral guidance cues. Binding of vascular endothelial growth factor-a (Vegfa) to its receptor, neuropilin-1 (Nrp1), has been demonstrated to support the growth of RGC axons at the midline^9^. Homophilic interactions between neuronal cell adhesion molecule (Nr-CAM) expressed in RGC axons and vHT cells have also been suggested to promote midline crossings of RGC axons^10^. Nr-CAM also cooperates with plexinA1, a receptor for semaphorin 6D (Sema6D), to support contralateral RGC axon projection^11^. However, a majority of RGC axons still cross the midline in mice lacking these cues, suggesting the presence of other key regulator(s) of contralateral RGC axon growth.

Ventral anterior homeobox 1 (Vax1) is expressed in the ventral and medial regions of the vertebrate forebrain^12,13^. The forebrain commissural structures, including the anterior commissure (AC), corpus callosum (CC), hippocampal commissure (HC), and OC, do not form properly in humans and mice with homozygous *VAX1* mutations^12,14,15^. Given the absence of *VAX1* gene expression in the commissural neurons, it had been thought that VAX1 functions as a transcription factor that induces the expression of axon growth factors in cells located along commissural axon growth tracks. However, it was found that the transcription factor activity of Vax1 is dispensable for the promotion of mouse RGC axon growth^16^. More surprisingly, Vax1 protein was detected in mouse RGC axons, despite the absence of autonomous *Vax1* gene expression in RGCs^16^. It was further found that Vax1 protein in mouse RGC axons is transferred from cells along RGC axon growth tracks and promotes axon growth by stimulating local mRNA translation^16^. However, whether intercellular Vax1 transfer is also critical for the growth of RGC axons in vivo remains unknown.

Here, we generated *Vax1*^*AA/AA*^ mice, in which *Vax1* was replaced by a transfer-defective *Vax1*^*AA*^ mutant. We found that the Vax1^AA^ protein was incapable of binding to heparan sulfate proteoglycans (HSPGs) and penetrating RGC axons, resulting in retarded growth of RGC axons. Most importantly, the OC was not formed in *Vax1*^*AA/AA*^ mutant mice, and RGC axons projected exclusively to ipsilateral brain areas. Consequently, the *Vax1*^*AA/AA*^ mice exhibited abnormal visuomotor responses, suggesting the importance of bilateral RGC axon growth in mice. Our findings in *Vax1*^*AA/AA*^ mice, therefore, not only confirm the physiological importance of Vax1 transfer but also provide a molecular basis for the visuomotor anomalies seen in achiasmatic mammals.

## Materials and methods

### Mouse strains

*Vax1*^*−/−*^ and *Pax6 α-Cre* mice were reported previously^12,17^. *Gt(ROSA)26Sor*^*tm4(ACTB-tdTomato,-EGFP)Luo*^*/J* (*R26*^*tm4*^) and *Gt(ROSA)26Sor*^tm11(CAG-tdTomato*,-GFP*)Nat^*/J* (*R26*^*tm11*^) mouse strains were purchased from the Jackson Laboratory^18,19^.

*Vax1*^*AA/AA*^ mice were generated using the CRISPR/Cas9 system with a CRISPR RNA (crRNA) and a trans-activating crRNA (tracrRNA) in the following procedures. Two crRNAs were designed to target two nearby sites of the second exon of the *Vax1* gene, which included DNA sequences encoding K101 and R102 (Supplementary Fig. 1a). The sequences of the crRNAs were #1, 5-TGGATCTGGACCGGCCCAAG-3 and #2, 5-AAGGACGTGCGAGTCCTCTT-3. The synthetic single-stranded DNA oligonucleotide (ssODN) containing missense (KR-to-AA) and synonymous mutations, 5’-TCTCAGAGAGATTGAGCTGCCGAGCCAGCTCGGTTCTCTCCCGGCCCACCACGTATTGGCAACGCTGGAACTCCATCTCCAGCCTGTAGAGCTGCTCAGCTGTAAACGAGGTCCTGGTCGCCGCGGGCCGGTCCAGATCCAAGCCTTTGGGCAGGATGATTTCTCGGATAGACCCCTTGGCATCTAGGAAAGGG-3’ (IDT, Inc., USA), was used as donor DNA. We then injected the crRNA/tracrRNA duplex and ssODN together with Cas9 mRNA (Toolgen, Inc., Seoul, Korea) into the cytoplasm of one cell-stage *C57BL/6* *J* mouse embryos. To screen the founders carrying the KR-to-AA mutation in the *Vax1* gene, PCR was performed (Supplementary Fig. 1b). Then, the genomic regions spanning the mutated second exon of the founder mice were validated by direct-sequencing analysis (Bionics Co., Ltd., Seoul, Korea). We obtained three male *Vax1*^*+/AA*^ mice after analyzing 83 pups obtained from the 439 injected embryos. The offspring of the *Vax1*^*+/AA*^ mice were then backcrossed with wild-type *C57BL/6* *J* mice over 6 generations to eliminate unwanted off-target mutations introduced by CRISPR/Cas9. Primer information is provided in Supplementary Table 2. All experiments were performed according to the Korean Ministry of Food and Drug Safety (MFDS) guidelines for animal research. The protocols were certified by the Institutional Animal Care and Use Committee (IACUC) of KAIST (KA2010-17) and Yonsei University (A-201507-390-01). All mice used in this study were maintained in a specific pathogen-free facility of the KAIST Laboratory Animal Resource Center and Yonsei Laboratory Animal Research Center.

### Cell and explant culture

Human cervical cancer HeLa cells and human embryonic kidney (HEK) 293 T cells were cultured in Dulbecco’s Modified Eagle’s Medium (DMEM) supplemented with 10% fetal bovine serum (FBS). For the luciferase assay, HEK293T cells (10^5^) were transfected with 1 μg pCAGIG-V5 vectors encoding Vax1, Vax1^AA^, or Vax1(R152S) cDNA together with pGL3-Tcf7l2-luciferase (0.2 μg) and pCMV-β-gal (0.2 μg) reporter constructs. Luciferase activity in the transfected cells was measured at 24 h posttransfection and normalized to β-galactosidase activity to obtain the relative luciferase activity of the cells. To test the cellular penetration of the Vax1 protein, V5 peptides or V5-Vax1 proteins, which were purified from HEK293T cells, were added to the growth media (1.5 pmol/ml [final concentration]) of HeLa cells, which express GFP-Sdc2. The distribution of V5 peptides and V5-Vax1 proteins on the cell surface and inside the cells was examined by immunostaining with mouse anti-V5 and chicken anti-GFP antibodies.

Retinal explants were prepared as described previously^16^. Briefly, the retina was prepared from mouse embryos at E13 and mixed with collagen in DMEM supplemented with 10% FBS. The retinal explants in collagen were then cultured in neurobasal medium containing B27 supplement (Invitrogen Inc.) for 48 h to allow the axons to grow from the explants. 6X-Histidine peptides or Vax1-6X-His proteins, which were purified from E. coli, were then added to the culture medium (2 pmol/ml [final concentration]) of retinal explants. Alternatively, the retinal explants were placed next to collagen droplets containing HEK293 cells (10^5^ cells/droplet), which express Vax1 or Vax1^AA^. The lengths of retinal axons grown from the explants were measured before and after the treatments to determine the axon growth rate.

### Detection of Vax1 proteins in the growth medium

Heparin (10 mg/ml) was added to the growth medium of HEK293T cells (10^7^) expressing V5-Vax1 or V5-Vax1^AA^. Macromolecules including the proteins and lipids in the growth medium were precipitated by adding trichloroacetic acid (TCA; 20% final). The precipitates were washed with cold acetone three times and dissolved in 2X-SDS sample buffer for SDS‒PAGE followed by WB to detect Vax1 proteins released in the growth medium.

### Immunohistochemistry and in situ RNA hybridization

The distribution of proteins in HeLa and mouse embryonic cells was examined by immunostaining. Cultured cells were fixed in 4% paraformaldehyde (PFA) in phosphate-buffered saline (PBS) for 10 min at 36 h posttransfection. Sections of mouse embryos, eyes, and brain slabs were fixed in 4% PFA/PBS at room temperature for 2 h and then placed in a 20% sucrose/PBS solution at 4 °C for 16 h before embedding in OCT (optimal cutting temperature) medium for cryofreezing and cryosectioning.

The cells and sections were incubated in a blocking solution containing 0.2% Triton X-100, 5% normal donkey serum, and 2% bovine serum albumen (BSA) in PBS for 1 h. To stain the proteins in the cells, the samples were incubated with the indicated primary antibodies in blocking solution without 0.2% Triton X-100 at 4 °C for 16 h and then with the appropriate secondary antibodies conjugated with fluorophores. Immunofluorescence was subsequently analyzed using Olympus FV1000 and Zeiss LSM810 confocal microscopes. Antibody information is provided in Supplementary Table 1.

Distributions of mRNA of interest in the embryonic sections were detected by in situ hybridization (ISH) with digoxygenin (DIG)-labeled RNA probes and visualized by immunostaining with alkaline phosphatase (AP)-conjugated α-DIG followed by AP-mediated colorization, as described in a previous report^16^.

### Tissue clearing, light sheet microscopy, and 3D image reconstitution

To visualize tdTom* fluorescence signals, the embryos were cleared by stabilization under harsh conditions via intramolecular epoxide linkages to prevent degradation (SHIELD) method as described previously^20^. In brief, mouse embryos were serially incubated in SHIELD perfusion solution, SHIELD-OFF solution, and SHIELD-ON solution. The samples were then delipidated for 3–5 days at 47 °C in SDS clearing buffer, followed by washing at 37 °C in PBS containing 1% Triton X-100 and 0.02% sodium azide for 24 h. The delipidated samples were incubated in optical clearing solution/PBS (50:50) for incubation at RT for 12 h and then in optical clearing solution at RT for 12–24 h until the samples became transparent. The samples were embedded in 1.5% agarose in optical clearing solution for imaging with a Zeiss Lattice Lightsheet 7 microscope. Collected images were processed for stitching and 3D reconstruction with ZEN software (Zeiss) and then analyzed by the surface tool of IMARIS 9.3 software (Bitplane) to render tdTom* fluorescence signals. To quantify the fluorescence signal intensity of the tdTom*-labeled RGC axons across the optic disc head and hypothalamic midline, valid fluorescence spots were identified by background subtraction. The distance between each spot and the sagittal plane was calculated.

### Chromatin immunoprecipitation (ChIP) and PCR

Chromatin immunoprecipitation was performed as described previously^15^. E10.5 mouse embryonic heads were isolated and chopped into small pieces prior to incubation in 1% formaldehyde in PBS at room temperature for 10 min. The nuclei were isolated for immunoprecipitation with rabbit anti-Vax1 antibody or preimmune rabbit IgG. DNA fragments coprecipitated with the antibodies were purified by phenol/chloroform/isoamyl alcohol extraction, and 100 ng of these immunoprecipitated DNAs was used as a template for PCR amplification of the Pax6 α-enhancer.

### Electroretinogram (ERG)

Mice were either dark- or light-adapted for 12 h before ERG recording and anesthetized with 2,2,2-tribromoethanol (Sigma). After the pupils of the mice were dilated with 0.5% tropicamide, a gold-plated objective lens was placed on the cornea, and silver-embedded needle electrodes were placed at the forehead and tail. ERG recordings were performed using a Micron IV retinal imaging microscope (Phoenix Research Labs) and analyzed by Labscribe ERG software according to the manufacturer’s instructions. To obtain scotopic ERG a- and b-waves, a digital bandpass filter ranging from 0.3 to 1000 Hz and stimuli ranging from −2.2 to 2.2 log(cd·s m^−2^) were used. To yield photopic ERG a- and b-waves, a filter ranging from 2 to 200 Hz and a stimulus ranging from 0.4 to 2.2 log(cd·s m^−2^) with a 1.3 log(cd·s m^−2^) background were used.

### In vivo extracellular recording and data analysis

We performed in vivo extracellular recordings in the monocular V1 (bregma, −3.50 mm; lateral, 2.50 mm; depth, 0.70 mm) of Vax1^+/+^ and Vax1^AA/AA^ mice. Mice were anesthetized with urethane (2 g per kg body weight, intraperitoneal injection) and restrained in a custom-designed head-fixed apparatus. A small craniotomy with a diameter of ~0.5 mm was made over V1 of the left and right hemispheres, and we inserted a 32-channel silicon electrode (A1x32-Poly3–10 mm-50-177-CM32, Neuronexus) using a microdrive motorized manipulator (Siskiyou). After waiting 20~30 min for stabilization, we started recording visual responses by presenting a full-field flashing light 5 times to the left eye. The visual stimuli were presented at 10 Hz for 500 ms, 5 pulses of 50 ms duration, and a total of 30 trials through a gamma-corrected monitor. Extracellular signals were filtered between 500~5000 Hz at a 30 kHz sampling rate, amplified by a miniature digital head-stage (CerePlex μ, Blackrock Microsystems), and saved through a data acquisition system (CerePlex Direct, Blackrock Microsystems). We performed spike sorting using Klusters software (http://neurosuite.sourceforge.net/) and further analyzed the firing rates of isolated single units using MATLAB. We analyzed the z score of firing activity in each single unit from −1 to +2 s of the onset of the visual stimuli and plotted the peri-stimulus time histogram (PSTH) of the normalized activity. The firing rate change index (FR index) of individual cells was calculated using the following formula:

$$\begin{array}{l}{{FR}}\,{{index}} = \left( {{{mean}}\,{{z - score}}\,{{for}}\,{{1s}}\,{{after}}\,{{stimuli}}\,{{onset}} - {{mean}}\,{{z}} - {{score}}\,{{for}}\,{{1s}}\,{{before}}\,{{stimuli}}\,{{onset}}} \right)\\\qquad\qquad\quad{{/}} ({{mean}}\,{{z - score}}\,{{for}}\,{{1s}}\,{{after}}\,{{stimuli}}\,{{onset + mean}}\,{{z - score}}\,{{for}}\,{{1s}}\,{{before}}\,{{stimuli}}\,{{onset)}}\end{array}$$

### Light-dark chamber assay

The light-dark test apparatus was composed of light (21 cm(width, W) X 29 cm(depth, D) X 20 cm(height, H), 700 lux) and dark (21 cm(W) X 13 cm(D) X 20 cm(H), ~5 lux) chambers. The dark chamber was separated from the light chamber by an entrance in the middle wall (5 cm (W) X 8 cm (H)). Mice were introduced into the light chamber with their heads toward the opposite side of the dark chamber and allowed to freely explore the apparatus for 10 min. The amount of time spent in the light and dark chambers and the number of transitions were analyzed by Ethovision XT10 software (Noldus).

### Looming assay

The looming test was performed as described previously with some modifications^21^. Briefly, the behavioral arena was prepared with an open-top acrylic box (30 cm(W) X 30 cm(D) X 30 cm(H)), which contained a nest in the shape of a triangular prism (10 cm(W) X 12 cm(D) X 10 cm(H)). The looming disk was programmed as a black circle on a gray background, increasing its size from 2 degrees of visual angle to 20 degrees in 250 ms and maintained for 250 ms. The pattern was repeatedly presented 10 times with 500 ms intervals for each trial.

### Optomotor response (OMR) test

Mouse visual acuity was measured with the OptoMotry system (Cerebral Mechanics) as previously described^22^. Mice were adapted to ambient light for 30 min and then placed on the stimulus platform, which was surrounded by four computer monitors displaying black and white vertical stripe patterns. An event in which mice stopped moving and began tracking the stripe movements with reflexive head turns was counted as a successful visual detection. The detection thresholds were then obtained from OptoMotry software.

### Measurements of pupillary contraction and optokinetic response (OKR)

Mouse heads were mounted to a plate and clamped to a holder to prevent head movement during measurement. Images of a mouse eye that showed the pupil and the corneal reflection were recorded by a CCD camera (120/240 Hz) with an infrared (IR) filter (ISCAN Inc.). To measure the OKR, the head-fixed mice were placed in front of screens that displayed a gray background or black and white vertical stripes (30% contrast) moving at a spatial frequency of 0.2 c/d and angular velocity of 12 d/s. To examine pupil contraction, the mice were kept in the dark for 30 s and then exposed to 500 lux of light for 10 s. The pupil position and diameter were measured by ISCAN software (ISCAN Inc.).

### Statistical analyses

Statistical tests were performed using Prism Software (GraphPad; v7.0) measurement tools. All data from statistical analysis are presented as the average ± STD. Comparison between two groups was done by unpaired Student’s t test, and the differences among multiple groups were determined by analysis of variance (ANOVA) with Tukey’s post-test used to determine the significant differences among multiple groups. P values (*p*) < 0.05 were considered statistically significant.

## Results

### Identification of a GAG sugar-binding motif in Vax1

Vax1 was found to be secreted from the cells that interact with developing mouse RGC axons and enters the axons. Binding of Vax1 to RGC axons was mediated by heparan sulfate (HS) sugar chains of HSPGs, such as syndecan (Sdc) and glypican (Glp)^16^. Another secreted homeodomain protein, orthodenticle homeobox 2 (Otx2), was also found to bind chondroitin sulfate (CS) sugar chains of chondroitin sulfate proteoglycan^23,24^. It was further identified that the binding of Otx2 to CS was mediated by the conserved glycosaminoglycan (GAG) binding motifs [-X-B-B-X-B-X-] and [-X-B-B-B-X-X-B-X-]^25^.

**Fig. 1:**
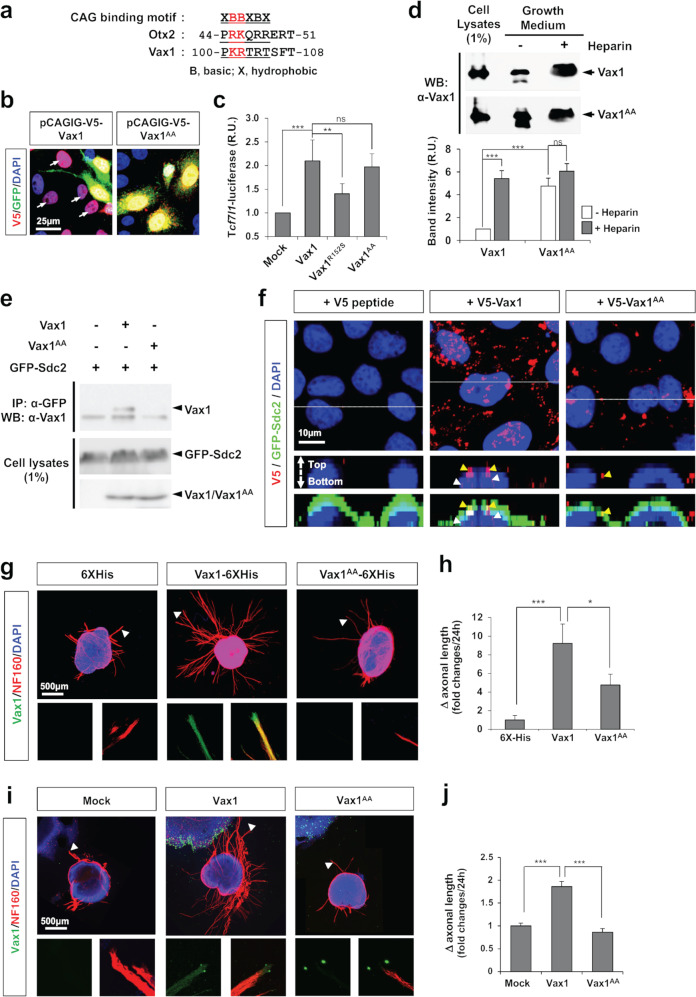
Identification of a GAG sugar-binding motif of Vax1. **a** Consensus amino acid sequences of GAG sugar-binding motifs in mouse Otx2 and Vax1. **b** Expression of V5-Vax1 and EGFP, which are independently translated from the same transcript, was examined by immunostaining transfected HeLa cells with mouse anti-V5 (red) and chicken anti-GFP (green) antibodies. Arrows indicate HeLa cells expressing V5-Vax1 without EGFP, implicating the transfer of V5-Vax1 but not EGFP from V5;EGFP double-positive cells. **c** HEK293T cells were transfected with a DNA vector encoding Vax1, Vax1(R152S), or Vax1(KR/AA) cDNA together with a Vax1 target *Tcf7l2*-luciferase reporter DNA construct. Luciferase activities in the transfected cells were measured at 24 h posttransfection. The values are averages obtained from four independent experiments, and error bars denote standard deviations (SDs). *P value*s were determined by ANOVA (*, *p* < 0.01; **, *p* < 0.005; ***, *p* < 0.001; ns, not significant). **d** V5-tagged Vax1 or Vax1^AA^ proteins were expressed in HEK293T cells, and growth media of the transfected cells were then collected after incubating for 3 h in the presence (+) or absence (-) of heparin (10 mg/ml final; see Methods for details). Cell lysates and TCA-precipitated fractions of growth medium were analyzed by 10% SDS‒PAGE and subsequent western blotting (WB) with anti-Vax1 antibody (α-Vax1). The graph below the WB data shows the relative intensities of the Vax1 bands in the blots. The values are averages obtained from four independent experiments, and error bars denote the SD. **e** Interactions of V5-Vax1 and V5-Vax1^AA^ with GFP-Sdc2 in HEK293T cells were assessed by immunoprecipitation (IP) with α-V5 and subsequent WB with α-GFP. Relative amounts of V5-Vax1 and GFP-Sdc2 in the cell lysates were also examined by WB. (**f**) V5-Vax1 or V5-Vax1^AA^ recombinant proteins were added to the growth medium of HeLa cells expressing GFP-Sdc2 and incubated for 3 h. Vax1 proteins inside cells and/or at the cell surface were detected by immunostaining with mouse α-V5 (red) and chick α-GFP (green). **g** Retinas were isolated from E13.5 mice and cultured as described in the Methods. The axonal lengths of retinal explants were measured at 24 h postculture; then, the explants were treated with 6X-His-tagged recombinant Vax1 or Vax1^AA^ proteins. (**i**) Alternatively, retinal explants were cocultured with HEK293T cells transfected with pCAGIG (mock), pCAGIG-V5-Vax1, or pCAGIG-V5-Vax1^AA^. Axonal lengths were remeasured after 24 h, and the explants were immunostained with α-Vax1 (green) and α-NF160 (red). Arrowheads indicate the areas magnified in each inset. **h** and **j** The changes in axonal length during the 24-h incubation period are shown in graphs. The values in the graph are averages, and error bars denote SDs (*n* = 6).

We identified that mouse Vax1 also contains a GAG-binding motif located at amino acids 100–105 (Fig. 1a). To investigate the possibility that Vax1 binds to HS chains through this putative GAG-binding motif, we replaced lysine and arginine (KR) amino acid residues in the motif with two alanines (A), yielding Vax1^AA^ (Fig. 1a). HeLa cells were transfected with a DNA construct that expresses an mRNA encoding *Vax1* or *Vax1*^*AA*^ together with enhanced green fluorescent protein (EGFP) but produces EGFP separately from Vax1 or Vax1^AA^ using an internal ribosome entry site (IRES). The KR-to-AA mutation suppressed Vax1 transfer from EGFP-positive donor cells to EGFP-negative recipient cells (Fig. 1b). However, it did not significantly affect Vax1 transcription factor activity, which was assessed by monitoring the expression of a luciferase reporter downstream of a Vax1 target sequence in the *transcription factor 7-like 2* (*Tcf7l2*) gene^26^ (Fig. 1c). This finding contrasts with the reduced transcription factor activity of the Vax1^R152S^ mutant (Fig. 1c), in which arginine 152 (R152) of the DNA binding motif was changed to serine (S)^14^.

We found that the level of Vax1^AA^ protein in the growth media of the transfected cells was significantly elevated compared with that of Vax1 (Fig. 1d). However, the amount of Vax1^AA^ in the medium was not further increased by the addition of free heparin, which competes with HS chains of HSPGs to bind extracellular Vax1 and release it from the HSPG-enriched cell surface into the growth medium^27^ (Fig. 1d). The results suggest reduced affinity of Vax1^AA^ for HS sugars compared with Vax1, together with a result that shows the impaired interaction between Vax1^AA^ and syndecan-2 (Sdc2) HSPG (Fig. 1e). Consequently, Vax1^AA^ added to the growth medium could bind or penetrate HeLa cells much less efficiently than Vax1 (Fig. 1f).

We next tested whether the KR-to-AA mutation also influences the binding and penetration of Vax1 to RGC axons and subsequent axon growth stimulation by Vax1. We found that Vax1^AA^ added to the growth medium of mouse retinal explants was not detected in neurofilament 160 (Nf160)-positive RGC axons, nor did it induce axonal growth as efficiently as Vax1, which penetrated retinal axons and significantly promoted axonal growth (Fig. 1g, h). We also found that Vax1^AA^ was not transferred from HEK293T cells to RGC axons, which were projected from the cocultured mouse retinal explants (Fig. 1i, j). Consequently, the lengths of RGC axons extending toward Vax1^AA^-expressing 293 T cells were shorter than those growing toward Vax1-expressing 293T cells. These results suggest that KR residues are necessary for the binding of Vax1 to HSPGs and subsequent penetration of Vax1 into RGC axons.

### Generation of Vax1^AA/AA^ mice

To investigate the consequences of the KR to AA mutation in vivo, we next introduced the corresponding mutation into the mouse *Vax1* gene to generate *Vax1*^*AA*^ mice (Supplementary Fig. 1a, b; see materials and methods for details). *Vax1*^*AA/AA*^ homozygous mice were born without any recognizable morphological defects and survived without significant health problems, whereas the mice carrying homozygous nonsense mutations (*Vax1*^*−/−*^) or hemizygous KR-to-AA mutations together with nonsense mutations (*Vax1*^*AA/−*^) died after birth with cleft palates that interfered with breathing^12^ (Supplementary Fig. 1c–e). Noticeably, the incisors grew continuously in approximately a quarter of *Vax1*^*AA/AA*^ mice (Supplementary Fig. 1d). The outgrowing incisors made it difficult for the mice to consume chow and caused lethality after weaning unless the outgrown incisors were cut regularly (Supplementary Fig. 1e).

**Fig. 2:**
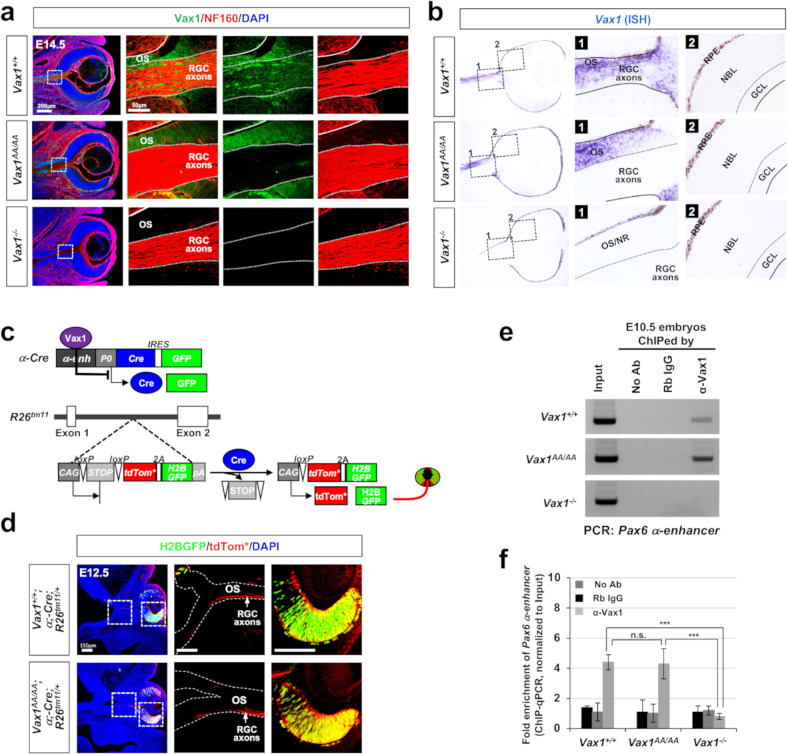
Vax1^AA^ exhibits defective intercellular transfer but intact transcription factor activity in vivo. **a** Intercellular transfer of Vax1 from E14.5 *Vax1*^*+/+*^ and *Vax1*^*AA/AA*^ littermate mouse OS cells to RGC axons was determined by immunostaining of the embryonic sections with α-Vax1 (green) and α-NF160 (red). **b** Expression of *Vax1* and *Vax1*^*AA*^ mRNA in E14.5 *Vax1*^*+/+*^ and *Vax1*^*AA/AA*^ littermate mouse eyes and OS was examined by ISH. Boxed areas in the leftmost column are magnified in two right columns. The specificities of the anti-Vax1 antibody (a) and *Vax1* ISH probe (b) were determined by the absence of signals in E14.5 *Vax1*^*−/−*^ mice. The *ISH* signals of the sense probes for *Vax1* did not exhibit specific signals (data not shown). The solid lines in the images indicate the boundary of OS, and the dotted lines mark the border between the OS and RGC axon bundles. NR, neural retina; RPE, retinal pigment epithelium; NBL, neuroblast layer; GCL, ganglion cell layer. **c** The activities of a Vax1 target *Pax6 α*-enhancer in mouse embryos were measured by detecting H2B-EGFP, the expression of which is induced together with membrane-bound tdTomato (tdTom*) at the *R26*^*tm11*^ gene locus after α-Cre-dependent excision of the *loxP-STOP-loxP* cassette. Arrowheads point to the frontlines of the RGC axons. **d** Binding of Vax1 and Vax1^AA^ proteins to the *Pax6 α*-enhancer was determined by PCR detection of *Pax6 α*-enhancer sequences in DNA fragments isolated from E10.5 mouse embryonic cells by ChIP with the indicated antibodies (see details in Methods). Input, mouse chromosomal DNA; No Ab, no antibody; Rb IgG, preimmune rabbit IgG; α-Vax1, rabbit anti-Vax1 polyclonal antibody. **e** Relative levels of *Pax6 α*-enhancer sequences in the ChIPed samples were compared by qPCR. **f** The numbers on the y-axis are the average 2^-ΔCt^ values of the samples against the critical threshold (Ct) values of the input. Error bars denote SD (*n* = 5).

Using *Vax1*^*AA/AA*^ mouse embryos, we examined whether the mutation affects the intercellular transfer of Vax1 in vivo. We found that Vax1^AA^ was strongly expressed in ventral medial forebrain structures, including the optic stalk (OS), of *Vax1*^*AA/AA*^ mouse embryos at 14.5 days post coitum (dpc; E14.5), a distribution pattern similar to that of Vax1 in *Vax1*^*+/+*^ littermate mice (Fig. 2a). Unlike Vax1, which was present in RGC axons as well as OS cells in *Vax1*^*+/+*^ mice, Vax1^AA^ was not detectable in the RGC axons in *Vax1*^*AA/AA*^ mice (Fig. 2a). However, *Vax1*^*AA*^ mRNA was present in the OS cells of *Vax1*^*AA/AA*^ mice (Fig. 2b), suggesting that the KR-to-AA mutation did not affect *Vax1* expression in OS cells but interfered with Vax1 protein transfer from OS cells to RGC axons.

### Intact transcription factor activity of Vax1^AA/AA^ in vivo

We examined whether the KA-to-AA mutation alters Vax1 transcription factor activity in vivo by monitoring the expression of a reporter driven by the *Pax6* α-enhancer, where Vax1 binds to suppress enhancer activity in OS cells^15^. In practice, we used *α-Cre;R26*^*tm11*^ mice^17,19^, which express histone 2B-GFP (H2B-GFP) and membrane-bound tdTomato (tdTom*) reporters at the *ROSA26* gene locus upon the excision of the *loxP-STOP-loxP* (*LSL*) cassette by Cre recombinase expressed downstream of the *Pax6* α-enhancer (Fig. 2c). The H2B-GFP signals were observed only in the nuclei of retinal cells but not in the OS cells of E12.5 *Vax1*^*+/+*^ mice, whereas the tdTom* signals were detectable in RGC axons in the OS as well as the retinal cells (Fig. 2d, top row). The pattern was not changed in their *Vax1*^*AA/AA*^ littermates (Fig. 2d, bottom row). The results, therefore, suggest that *Pax6* α-enhancer activity was properly suppressed in the OS cells of *Vax1*^*+/+*^ and *Vax1*^*AA/AA*^ mice.

We also compared the abilities of Vax1 and Vax1^AA^ to bind *Pax6* α-enhancer sequences in vivo. Vax1-bound DNA fragments were isolated from E10.5 mouse heads by chromosome immunoprecipitation (ChIP) using an anti-Vax1 antibody and used for polymerase chain reaction (PCR) and quantitative PCR (qPCR) detection of *Pax6* α-enhancer sequences in the ChIPed DNA. The results revealed no significant difference in the binding abilities of Vax1 and Vax1^AA^ to the target sequences (Fig. 2e, f). Together, these results suggest that the changes in *Vax1*^*AA/AA*^ mice compared with *Vax1*^*+/+*^ mice are unlikely to result from the alteration of Vax1 target gene expression in cells with active *Vax1* gene expression.

### Developmental delay of the OS in Vax1^AA/AA^ mice

**Fig. 3:**
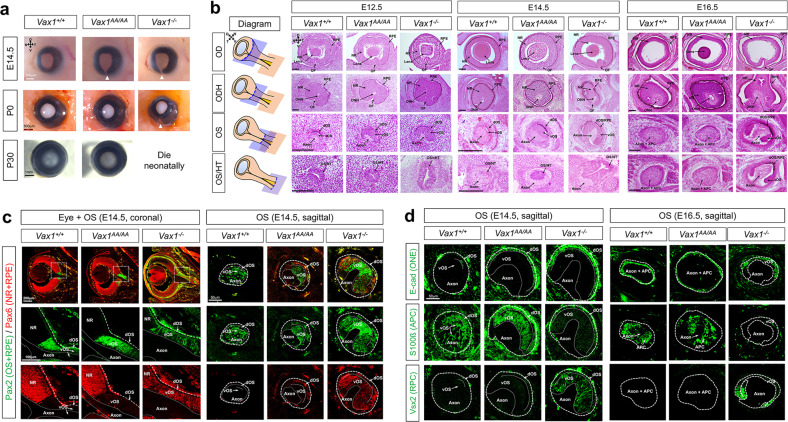
Developmental delay of OS differentiation in *Vax1*^*AA/AA*^ mice. **a** Pictures of mouse eyes (frontal view) with the indicated genotypes are taken at various developmental stages. Arrowheads point to the optic fissures. N, nasal; T, temporal; D, dorsal; V, ventral. **b** Sagittal sections of mouse embryos were stained with hematoxylin & eosin (H&E). Positions of the sections are indicated by blue plates in the diagram (leftmost column). APC, astrocyte precursor cell; ODH, optic disc head; OF, optic fissure; dOS, dorsal optic stalk; vOS, NR, neural retina; RPE, retinal pigment epithelium; ventral optic stalk; vHT, ventral hypothalamus. Scale bars in the pictures are 100 μm. **c** The distributions of Pax6 and Pax2 in E14.5 mouse embryonic sections were examined by immunostaining. Boxed areas in top row images of coronal sections are magnified in two bottom rows. **d** Sagittal sections including the medial OS of E14.5 and E16.5 mouse embryos were stained with antibodies that recognize the corresponding markers. APC, astrocyte precursor cell; ONE, optic neuroepithelium; RPC, retinal progenitor cell.

Next, we investigated the phenotypic alterations of *Vax1*^*AA/AA*^ mice in comparison to *Vax1*^*+/+*^ and *Vax1*^*−/−*^ mice. Coloboma, the fissures in the ventral eyecup and OS, is observed in the eyes of humans and mice harboring *VAX1* mutations^12–14^. A coloboma was not observed in neonatal (i.e., postnatal Day 0, P0) *Vax1*^*AA/AA*^ mouse eyes, unlike in *Vax1*^*−/−*^ mouse eyes (Fig. 3a [middle row], 3b; fissures in the colobomatous eyes are indicated by arrowheads). However, the optic fissures remained unclosed in *Vax1*^*AA/AA*^ mice by E14.5, similar to *Vax1*^*−/−*^ mouse eyes (Fig. 3a [top row], 3b). The unclosed OS in E14.5 *Vax1*^*AA/AA*^ mice expressed paired homeobox 2 (Pax2), an OS marker, but not Pax6, a retinal marker, similar to the distribution observed in *Vax1*^*+/+*^ littermates (Fig. 3c [coronal], left and center columns). This contrasts with E14.5 *Vax1*^*−/−*^ mice, which coexpressed Pax2 and Pax6 in the OS (Fig. 3c [coronal], right column). Therefore, these results suggest that OS fate is specified properly in *Vax1*^*AA/AA*^ mice but not in *Vax1*^*−/−*^ mice.

The Pax2-positive OS cells were, however, clustered separately from RGC axons in E14.5 *Vax1*^*AA/AA*^ mice, whereas they were spread among RGC axons in E14.5 *Vax1*^*+/+*^ mice (Fig. 3c, sagittal). The OS cells in E14.5 *Vax1*^*+/+*^ mice expressed an astrocyte precursor cell (APC) marker, S100 calcium-binding protein ß (S100ß), but lost a neuroepithelium marker, E-cadherin (E-cad) (Fig. 3d, left column of the E14.5 panel). The cells in the ventral OS (vOS) of E14.5 *Vax1*^*AA/AA*^ mice also expressed S100 without E-cad, while those in the dOS coexpressed S100ß and E-cad (Fig. 3d, center column of the E14.5 panel). The vOS cells in E14.5 *Vax1*^*−/−*^ mice, however, did not express S100ß; instead, they expressed the retinal marker Vsx2 (Fig. 3d, right column of the E14.5 panel). In E16.5 *Vax1*^*AA/AA*^ mice, similar to the distribution observed in *Vax1*^*+/+*^ littermates, S100ß-positive OS cells entirely lost E-cad expression and started to disperse between RGC axons, whereas S100ß-negative OS cells still formed Ecad-positive neuroepithelial clusters in E16.5 *Vax1*^*−/−*^ mice (Fig. 3d, E16.5 panel). These results suggest that the differentiation of S100ß-positive APCs from the OS neuroepithelium is impaired in *Vax1*^*−/−*^ mice but merely delayed in *Vax1*^*AA/AA*^ mice.

### Growth retardation of RGC axons in the OS of Vax1^AA/AA^ mice

**Fig. 4:**
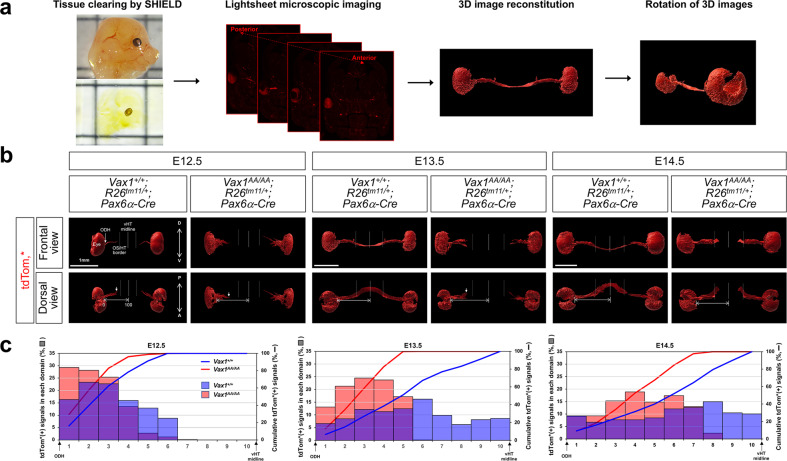
Retarded RGC axon growth in *Vax1*^*AA/AA*^ mice. **a** Schematic diagram depicting the 3D reconstitution of tdTom* fluorescent signals in RGC axons. Retinal cells, including RGCs, in *Vax1*^*+/+*^ and *Vax1*^*AA/AA*^ littermate mouse embryos, which were cleared by the SHIELD method, were visualized by the tdTom* reporter expressed in the *R26*^*tm11*^ gene locus in a Pax6 α-Cre-dependent manner. The tdTom* signals of retinal cells and RGC axons, which grow in the OS to form the OC prior to extending through the OT, were then reconstituted in 3D (see details in Methods). **b** The 3D reconstituted images of E12.5, E13.5, and E14.5 mouse embryos are shown. **c** Relative tdTom* intensities in each domain of the RGC axon track, which is divided into 10 segments from the optic nerve head (OHD) to the vHT midline, are shown in the graphs. Cumulative tdTom* intensities, which represent the positions of RGC axon terminals, are also shown in the graphs. The values are averages (*n* = 3; 3 independent litters).

We also examined whether RGC axons could grow in *Vax1*^*AA/AA*^ mouse OS as fast as those in *Vax1*^*+/+*^ mouse OS. To visualize RGC axons in 3 dimensions (3D), we reconstituted the tdTom* fluorescence reporter signals of tissue-cleared *Vax1*^*+/+*^*;α-Cre;R26*^*tm11*^ and *Vax1*^*AA/AA*^*;α-Cre;R26*^*tm11*^ mouse embryos after light sheet microscopic imaging (Fig. 4a; see details in Materials and methods). On E12.5, RGC axons were observed commonly in the OS of *Vax1*^*+/+*^ and *Vax1*^*AA/AA*^ littermate mice (Fig. 4b [left panel]; Supplementary videos 1 and 2), indicating that RGC axons exit from the eyes properly in *Vax1*^*AA/AA*^ mice. However, the lengths of RGC axons in E12.5 *Vax1*^*AA/AA*^ mice were shorter than those of *Vax1*^*+/+*^ littermates (Fig. 4c). The RGC axon terminals were still observed in the OS of E13.5 *Vax1*^*AA/AA*^ mice, while those had passed the OS/HT borders and reached the vHT midline in *Vax1*^*+/+*^ littermates (Fig. 4b [center panel], 4c; Supplementary video 3 and 4). The length of RGC axons in E14.5 *Vax1*^*AA/AA*^ mice remained shorter than those of *Vax1*^*+/+*^ littermate mice (Fig. 4b [right panel]; Supplementary videos 5 and 6). Moreover, RGC axons were absent in the vHT midline but found in the lateral HT wall in E14.5 *Vax1*^*AA/AA*^ mice, whereas the axons extended underneath the vHT to form the OC in E14.5 *Vax1*^*+/+*^ littermates. The numbers of Brn3b-positive RGCs in E14.5 *Vax1*^*+/+*^ and *Vax1*^*AA/AA*^ mouse retinas were not significantly different from each other (Supplementary Fig. 2), suggesting that the weak axon signals in *Vax1*^*AA/AA*^ mouse OS did not result from the decrease in RGC numbers. Together, these results suggest that RGC axons grow slowly in the OS to arrive at the HT late and then do not access the vHT to cross the midline but extend ipsilaterally in *Vax1*^*AA/AA*^ mice.

Despite the growth retardation of RGC axons, *Netrin-1*, an RGC axon growth factor^28^, was found in the OS of *Vax1*^*AA/AA*^ mice, whereas it disappeared in *Vax1*^*−/−*^ mouse OS (Supplementary Fig. 3a, b). The expression of Semaphorin-5A (Sema5A), which is a repulsive RGC guidance cue expressed in OS cells^29^, was also expressed properly in the OS of *Vax1*^*+/+*^ and *Vax1*^*AA/AA*^ mice but not in *Vax1*^*−/−*^ mouse OS (Supplementary Fig. 3c, d). The results suggest that retarded RGC axon growth in *Vax1*^*AA/AA*^ mouse OS did not result from the altered expression of these RGC axon guidance cues.

### Structural alteration of the vHT in achiasmatic Vax1^AA/AA^ mouse embryos

**Fig. 5:**
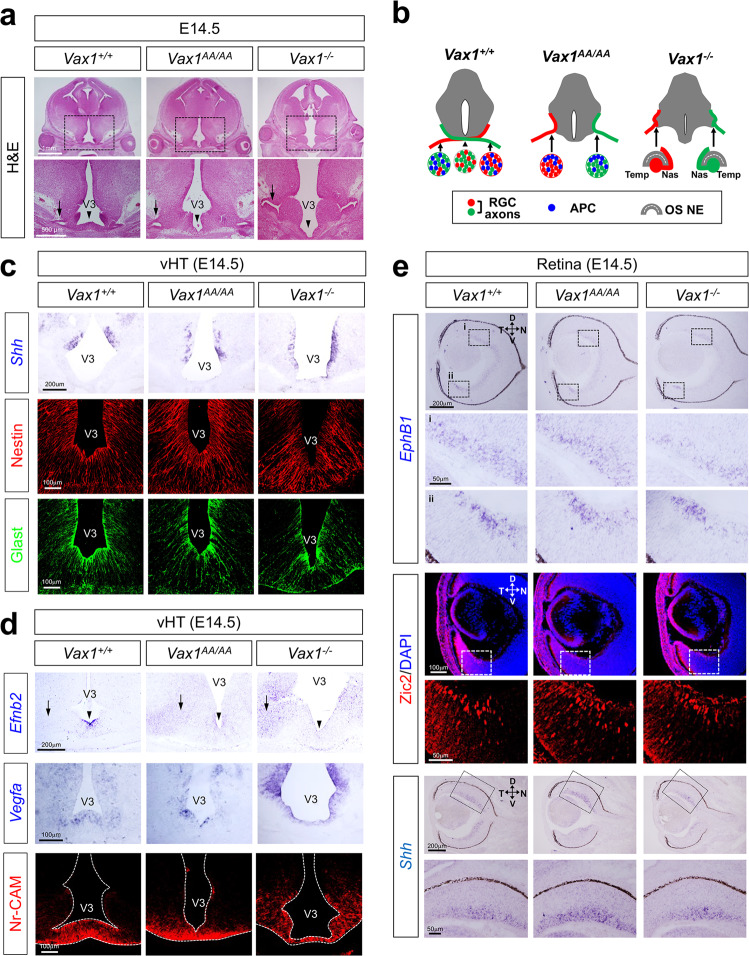
Expression of RGC axon guidance cues in *Vax1*^*AA/AA*^ mice. **a** Coronal sections of E14.5 mouse embryos were stained with H&E. Arrows indicate the OS/HT junction, and arrowheads indicate the vHT midline. V3, third ventricle. **b** Schematic diagrams that show the structures of OS/HT junctions and RGC axon pathways in *Vax1*^*+/+*^, *Vax1*^*AA/AA*^, and *Vax1*^*−/−*^ mice. Nas, nasal; Temp, temporal. OS NE, OS neuroepithelium. (**c**) Distributions of *Shh* mRNA in embryonic sections were detected by in situ hybridization (ISH). Development of hypothalamic radial glia (RG) from NPCs was determined by immunostaining for the RG marker Glast (glutamate aspartate transporter 1) and the NPC marker Nestin. **d** Expression of a repulsive guidance cue, ephrinB2, and the attractive guidance cues Vegfa and Nr-CAM, for RGC axons in E14.5 mouse vHT was examined by *ISH* (*Efnb2* and *Vegfa*) and IHC (Nr-CAM). Arrows indicate the OS/HT junction, and arrowheads indicate the vHT midline. **e** Expression levels of retinal genes that induce ipsilateral RGC axon projection were examined by ISH (*EphB1* and *Shh*) and IHC (Zic2). Boxed areas in top row images are magnified in bottom rows. The *ISH* signals of the sense probes for *Efnb2*, *Vegfa*, *Ephb1*, and *Shh* did not exhibit specific signals (data not shown).

We found that the HT in *Vax1*^*AA/AA*^ and *Vax1*^*−/−*^ mouse embryos protruded ventrally without underlying RGC axon bundles, while the vHT of E14.5 *Vax1*^*+/+*^ mice were flattened over RGC axons forming the OC (Fig. 5a, b). However, *Shh*, which is critical for specification of the vHT and OS^30,31^, showed a similar expression pattern in E14.5 *Vax1*^*+/+*^, *Vax1*^*AA/AA*^ and *Vax1*^*−/−*^ mice (Fig. 5c, top row). This suggests that fate specification of the vHT is likely unaffected in *Vax1*^*AA/AA*^ and *Vax1*^*−/−*^ mice, as has been shown in previous reports^12,13^. Glast-positive radial glia, which express various RGC axon guidance cues^4,5^, were also observed in the vHT of *Vax1*^*AA/AA*^ and *Vax1*^*−/−*^ mice, indicating normal development of vHT radial glia from Nestin-positive neuroepithelium (Fig. 5c, second and third rows).

We thus examined whether the altered expression of RGC axon guidance cues in the vHT is related to the failure of RGC axon growth toward the vHT midline in *Vax1*^*AA/AA*^ and *Vax1*^*−/−*^ mice. However, attractive guidance cues for RGC axons, including Vegfa and Nr-Cam^9,10^, were expressed properly in the vHT of E14.5 *Vax1*^*AA/AA*^ and *Vax1*^*−/−*^ mice (Fig. 5d, second and third rows). The expression of *Ephrin-B2* (*Efnb2*), which triggers the repulsion of EphB1-expressing RGC axons^7^, was decreased in the vHT midline cells of E14.5 *Vax1*^*AA/AA*^ and *Vax1*^*−/−*^ mice in comparison to E14.5 *Vax1*^*+/+*^ littermates’ vHT midline glia (Fig. 5d, arrows in top row; Supplementary Fig. 4). Instead, *Efnb2* mRNA was detectable at low levels in the lateral HT area in E14.5 *Vax1*^*AA/AA*^ and *Vax1*^*−/−*^ mice (Fig. 5d, arrowheads in top row). However, given no repulsion of the majority of RGC axons, except for a minority of *EphB1*-high RGC axons from the VT retina, by the vHT midline glia expressing *Efnb2* at high levels^7^, the low level of *Efnb2* in the lateral HT of *Vax1*^*AA/AA*^ mice should be insufficient to trigger repulsive responses in all RGC axons.

We also found that the expression levels of *EphB1* and its upstream regulator Zic2 were enriched in the VT RGCs of E14.5 *Vax1*^*AA/AA*^ and *Vax1*^*−/−*^ mouse retinas, similar to the expression patterns in E14.5 *Vax1*^*+/+*^ mice (Fig. 5e)^32^. These results imply that there might be no ectopic activation of EphB1 in *Vax1*^*AA/AA*^ and *Vax1*^*−/−*^ mouse RGC axons. Shh, which was identified as a transacting repulsive cue in mouse RGC axons^8^, was also expressed properly in RGCs of those mice (Fig. 5e). Together, our data suggest that *Vax1*^*AA/AA*^ mouse RGC axons might be repelled from the HT by signals other than Efnb2 and Shh. It is also possible that RGC axons in *Vax1*^*AA/AA*^ mice could not access the vHT midline by losing axon growth signals other than Vegfa and Nr-Cam.

### Ipsilaterally biased retina‒brain connections in the Vax1^AA/AA^ mice

**Fig. 6:**
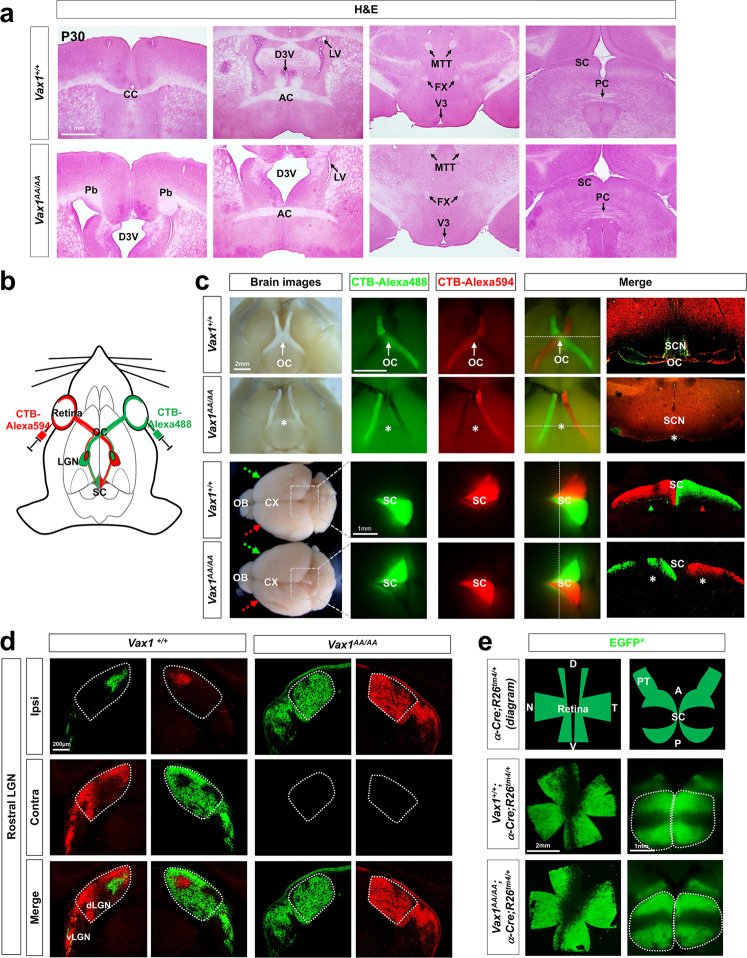
Ipsilaterally biased RGC axon projection in *Vax1*^*AA/AA*^ mice. **a** Coronal sections of P30 mouse brains were stained with H&E. The sections containing the indicated commissural structures were identified and shown. AC, anterior commissure; CC, corpus callosum; D3V, dorsal third ventricle; FX, fornix; Pb, probus bundle; PC, posterior commissure; SC, superior colliculus; MTT, mammillo-thalamic tract. **b** Diagram depicting the fluorescence labeling of mouse RGCs. (**c**) Alexa 488- and Alexa 594-labeled CTB proteins were injected into the right and left eyes of the mice at P28, respectively. Brains of the CTB-injected mice were isolated at P30, and fluorescent signals emitted from the brains were visualized by an Axio Zoom stereoscope (Zeiss; three center columns). Then, the fluorescent signals in coronal sections of the brains were detected by an FV1000 confocal microscope (Olympus; rightmost column). Bright-field images of the brains were also taken to show the structure of the brains and optic nerves (leftmost column). CX, cerebral cortex; OB, olfactory bulb; *, ventral midline of HT equivalent to the position of the OC in *Vax1*^*+/+*^ littermates. **d** Coronal sections of the CTB-injected mouse brains were collected, and fluorescent signals in the LGN area were detected. The areas surrounded by dotted lines are the dLGN. Contra, contralateral LGN section; Ipsi, ipsilateral LGN section. **e** The retinas and brains of P30 *Vax1*^*+/+*^ and *Vax1*^*AA/AA*^ littermate mice carrying α-*Cre*;*R26*^*tm4/+*^ transgenes, which express membrane-targeted EGFP (EGFP*) at the *R26*^*tm4*^ gene locus upon Cre-dependent excision of the loxP-tdTomato*-loxP gene cassette, were isolated and visualized. The areas surrounded by dotted lines are SC. PT, pretectum.

Using adult *Vax1*^*AA/AA*^ mice, we were able to analyze the structures and functions of the mouse visual system, which are difficult to study in *Vax1*^*−/−*^ mice because they die perinatally^12,13^. We found no significant difference in brain size and shape between P30 *Vax1*^*+/+*^ and *Vax1*^*AA/AA*^ littermate mice (Fig. 6a, c [left column]). The olfactory bulb (OB) of *Vax1*^*AA/AA*^ mice also appeared normal (Fig. 6c, the image in left bottom corner), although a previous report noted hypoplastic OBs in the few surviving *Vax1*^*−/−*^ mice^33^. Furthermore, *Vax1*^*AA/AA*^ mice have only one pituitary gland (data not shown), whereas *Vax1*^*−/−*^ mice have an extra pituitary gland by virtue of failure to suppress ectopic pituitary fate specification in the ventral anterior forebrain^34^. These results also suggest that Vax1-dependent specification of ventral forebrain structures is not significantly altered in *Vax1*^*AA/AA*^ mice.

Among the commissures that are reported to be absent in *Vax1*^*−/−*^ mice^12^, the CC and OC are missing in *Vax1*^*AA/AA*^ mice, whereas the AC and fornix are present (Fig. 6a, c [left column]). The posterior commissure (PC) was also present and appropriately positioned beneath the SC in *Vax1*^*AA/AA*^ mice (Fig. 6a, rightmost column). Furthermore, we visualized the RGC axons of mice using fluorescent dye-conjugated cholera toxin B (CTB) protein^35^. Alexa Fluor 488-conjugated CTB and Alexa Fluor 594-conjugated CTB were injected into the right and left eyes, respectively, of *Vax1*^*+/+*^ and *Vax1*^*AA/AA*^ littermate mice at postnatal Day 28 (P28; Fig. 6b). Fluorescence signals emitted by CTB-labeled RGC axons in the mice were then detected at P30 (Fig. 6c). Green and red fluorescence signals were predominantly detected in RGC axons that projected across the midline in *Vax1*^*+/+*^ mice (Fig. 6c, top row). Consequently, the majority of CTB fluorescence signals were observed in the contralateral SCs of *Vax1*^*+/+*^ mice (Fig. 6c, third row). In contrast, fluorescence signals of CTB were detected exclusively in the ipsilateral SCs of *Vax1*^*AA/AA*^ mice, in which it was not possible to detect the OC where differentially labeled RGC axons met (Fig. 6c, second and bottom rows).

We also examined whether ipsilaterally projecting *Vax1*^*AA/AA*^ mouse RGC axons were properly connected to the dLGN, a thalamic nucleus that relays visual information from the retina to the visual cortex^1^. Axons from RGCs in the ventral-temporal mouse retina project to the ipsilateral dLGN, where the majority of RGC axons from the contralateral retina are connected^4,5^. The minor ipsilateral axon terminals are then segregated from the major contralateral axon terminals by a retinal activity-dependent refinement process during the postnatal period^36,37^. Segregation of binocular RGC axons was clearly seen in the dLGN of P30 *Vax1*^*+/+*^ mice (Fig. 6d, left two columns). However, RGC axons in the dLGN of P30 *Vax1*^*AA/AA*^ mice originated only from the ipsilateral retina, and no contralateral RGC axons were observed in the dLGN (Fig. 6d, right two columns).

We further examined whether retinocollicular topographic connectivity was established properly in the *Vax1*^*AA/AA*^ mouse SC, which lacks contralateral RGC axon terminals (Fig. 6c, bottom row). Axons from RGCs in the temporal retina are known to connect to the anterior SC, whereas those from the nasal retina link to the posterior SC^38^. In the perpendicular axis, RGC axons from the dorsal retina arrive at the lateral SC, and those from the ventral retina are wired to the medial SC. Thus, axons of Pax6 α-Cre-affected RGCs in the ventral and peripheral retina, which express membrane-localized EGFP (EGFP*) in the *ROSA26* gene locus of *R26*^*tm4*^ Cre reporter mice^17,18^, map to medial and peripheral SC areas in P30 *Vax1*^*+/+*^ mice (Fig. 6e, middle row). This pattern was also observed in the SCs of *Vax1*^*AA/AA*^ littermate mice (Fig. 6e, bottom row), implying that the retinocollicular topography is established properly in *Vax1*^*AA/AA*^ mice.

### Light-stimulated retinal and cortical responses in Vax1^AA/AA^ mice

**Fig. 7:**
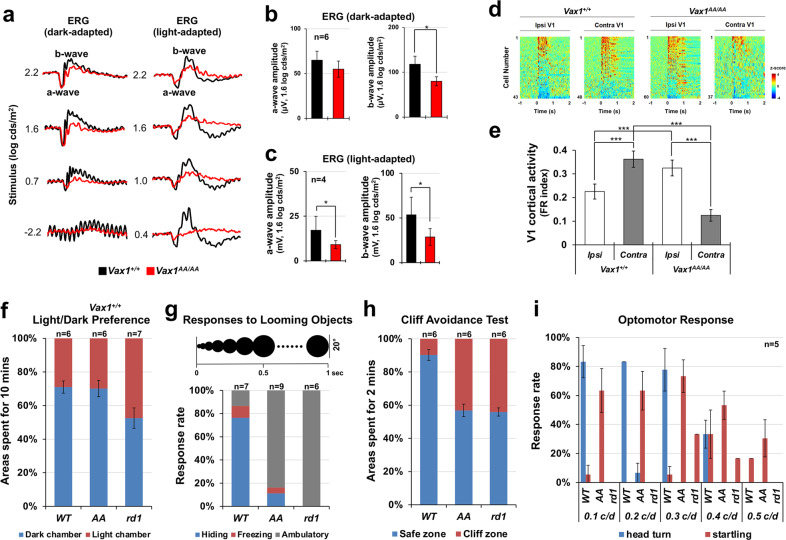
Reduced visual acuity of *Vax1*^*AA/AA*^ mice. **a** Electrophysiological activities of P45 *Vax1*^*+/+*^ and *Vax1*^*AA/AA*^ mouse retinas were examined by ERG (see details in Methods). The amplitudes of scotopic **b** and photopic **c** ERG a- and b-waves at the 1.6 log cds/m^2^ condition are presented. The numbers of mice tested are given in the graphs (4 independent litters). **d** Light-evoked excitation of cortical neurons in P45 *Vax1*^*+/+*^ and *Vax1*^*AA/AA*^ mouse V1 was measured by silicon multielectrode probes after monocular illumination (see details in Methods). Responses of neurons in the monocular zones of the ipsilateral (Ipsi) and contralateral (Contra) visual cortices were recorded. The color-coded heatmap represents the average z scores of spike firing rates. Red indicates an increase, and blue indicates a decrease in firing rates from the baseline. **e** Mean firing-rate change index of ipsilateral and contralateral V1 neurons in *Vax1*^*+/+*^ and *Vax1*^*AA/AA*^ mice. The y-axis values are the mean ± SEM. **f** Relative occupancy of light and dark chambers by P45 *Vax1*^*+/+*^ (*WT*), *Vax1*^*AA/AA*^ (*AA*), and *Pde6b*^*rd1/rd1*^ (*rd1*) mice for a 10-min measurement period was determined and shown in a graph. **g** Escaping responses of the mice, which were given the expanding black circle on top screens to mimic a looming shadow of a predator, were determined and shown in a graph. **h** To determine the stereoscopic vision of the mice, the relative occupancy of safe and cliff zones by the mice for the 2-min measurement period was determined and is shown in a graph. (**i**) The accuracy of the mice in turning their heads in the directions where black and white vertical stripes rotate was measured and shown in a graph (head turn). Startling responses of the mice in response to the stimuli were also measured (startling). The numbers of mice tested are given in the graphs in f – h.

Next, we examined whether visual information can be processed in the retina and delivered to the brain in achiasmatic *Vax1*^*AA/AA*^ mice. First, we tested the activities of mouse retinas using electroretinography (ERG) recordings. The shapes of ERG a-waves of dark-adapted P45 mouse retinas, which reflect the function of rod photoreceptors, appeared normal in *Vax1*^*AA/AA*^ mice, although the amplitudes of ERG b-waves, which are generated by bipolar cells and Müller glia in the inner retina downstream of photoreceptors^39^, were decreased slightly (Fig. 7a [right column], b). In contrast, the amplitudes of ERG a-waves of light-adapted mouse retinas, which reflect the activity of cone photoreceptors, were decreased significantly in *Vax1*^*AA/AA*^ mice compared with *Vax1*^*+/+*^ littermate mice (Fig. 7a [left column], c [graph in left]), leading to a consequent decrease in amplitudes of ERG b-waves (Fig. 7a [left column], c [graph in right]). The reduced photopic ERG responses in *Vax1*^*AA/AA*^ mice were likely related to the decrease in S-opsin–positive cone photoreceptors in the ventral retinas of *Vax1*^*AA/AA*^ mice, which did not show significant differences in other cell types in the retina and optic nerves (Supplementary Fig. 5).

We also examined whether visual information received by the retina is delivered to the visual cortex via the dLGN in *Vax1*^*AA/AA*^ mice. To this end, we recorded electrical activities of neurons in the monocular zone of the primary visual cortices (V1) in both hemispheres of the mouse brain while presenting the visual stimuli only to the left eye. We found that this predominantly triggered responses of cortical neurons in the right (i.e., contralateral) V1 of *Vax1*^*+/+*^ mice; conversely, it mainly activated neurons in the left (i.e., ipsilateral) V1 of *Vax1*^*AA/AA*^ mice (Fig. 7d, e). These results demonstrate that the *Vax1*^*AA/AA*^ mouse retina sends signals to the ipsilateral dLGN for subsequent delivery of information to the visual cortex, whereas retinal signals are sent mainly to the contralateral routes in *Vax1*^*+/+*^ mice.

### Reduced visual acuity of Vax1^AA/AA^ mice

We next examined whether the ipsilaterally biased retinogeniculate and retinocollicular pathways influenced the visual responses of *Vax1*^*AA/AA*^ mice. First, we tested whether the mice could discriminate between light and dark spaces. We found that P45 *Vax1*^*+/+*^ and *Vax1*^*AA/AA*^ littermate mice spent longer periods in the dark chamber than in the illuminated chamber (Fig. 7f). In contrast, P45 retinal dystrophy 1 (*rd1*) mutant *C3H/HeJ* blind mice, which have a homozygous mutation of the *phosphodiesterase 6b* (*Pde6b; Pde6b*^*rd1/rd1*^*)* gene^40^, spent equivalent periods in dark and light chambers (Fig. 7f). These results suggest that *Vax1*^*AA/AA*^ mice discriminate between light and dark as efficiently as *Vax1*^*+/+*^ mice.

Second, we examined whether mice could recognize images that mimic a looming shadow of predator^21^. P45 mice were placed in a transparent box covered by a computer monitor displaying a black circle that expands by a 20^°^ angle from the mouse head. *Vax1*^*+/+*^ mice froze or hid under a shelter while the circle in the monitor expanded (Fig. 7g; Supplementary video 7). *Vax1*^*AA/AA*^ mice also exhibited hiding and/or freezing responses; however, their response frequency was significantly lower than that of *Vax1*^*+/+*^ mice (Fig. 7g; Supplementary video 8). *Pde6b*^*rd1/rd1*^ mice showed no behavioral responses at all (Fig. 7g; Supplementary video 9). These results suggest that *Vax1*^*AA/AA*^ mice could recognize the shadow pattern but did so less efficiently than *Vax1*^*+/+*^ mice.

Third, we investigated whether mice could discriminate near and far objects by placing them on a transparent plate, half of which was printed with a flannel pattern image (i.e., safe zone), and the other unprinted half was extended over a floor printed with the same flannel pattern (i.e., cliff zone)^41^. P45 *Vax1*^*+/+*^ mice stayed in the safe zone and rarely ventured into the cliff zone (Fig. 7h; Supplementary video 10). However, *Vax1*^*AA/AA*^ littermate mice moved freely between the cliff and safe zones (Fig. 7h; Supplementary video 11), as did P45 *Pde6b*^*rd1/rd1*^ blind mice (Fig. 7h; Supplementary video 12). These results suggest that *Vax1*^*AA/AA*^ mice might have impaired depth perception.

Finally, we also assessed the visual acuity of mice by measuring optomotor responses (OMRs) to horizontally moving black and white vertical strips using OptoMotry^22^. P45 *Vax1*^*+/+*^ mice turned their heads in the direction of vertical stripe movement (Fig. 7i; Supplementary video 13); however, P45 *Vax1*^*AA/AA*^ and *Pde6b*^*rd1/rd1*^ mice failed to show a valid OMR (Fig. 7i; Supplementary video 14 and 15). These results suggest that the visual acuity of *Vax1*^*AA/AA*^ mice is significantly compromised compared with that of *Vax1*^*+/+*^ mice.

### Seesaw nystagmus of Vax1^AA/AA^ mice

The OMR visual acuity test counts head-turn events to moving objects. Therefore, mice may not respond properly if their oculomotor system is abnormal. Interestingly, *Vax1*^*AA/AA*^ mice startled in response to the stimuli, although they did not show corresponding head-turn behaviors (Fig. 7i; Supplementary video 14). This contrasted with *Pde6b*^*rd1/rd1*^ blind mice, which showed no stimulus-dependent responses (Fig. 7i; Supplementary video 15). These results suggest that the reduced visual acuity of *Vax1*^*AA/AA*^ mice might have resulted from an abnormal visuomotor system that cannot trigger proper head-turn responses.

**Fig. 8:**
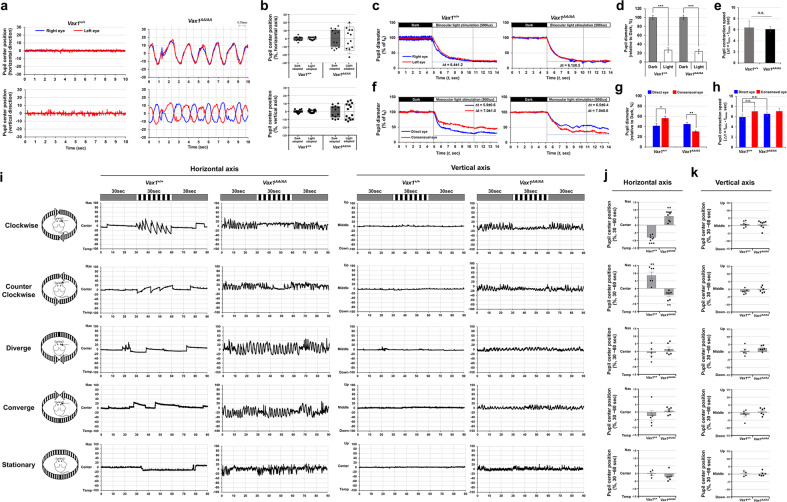
Visuomotor anomalies of *Vax1*^*AA/AA*^ mice. **a** Positions of pupil centers in the right and left eyes of head-fixed P45 *Vax1*^*+/+*^ and *Vax1*^*AA/AA*^ mice were tracked by the iSCAN rodent eye tracking system while the mice were kept in the dark. The relative positions of the pupil centers against the position at time 0 (t_0_) were plotted in the oscillograms. **b** Peak positions that the pupil centers moved at vertical and horizontal axes were measured, and the averages are shown in graphs. Error bars are SDs (*n* = 6). **c** The P45 mice were adapted to the dark for 30 min and illuminated with a room light. **f** Alternatively, the dark-adapted mice were illuminated monocularly with a point light. Pupil diameters were measured by an iSCAN eye tracking system before and after the illuminations. Relative pupil diameters against t_0_ are plotted in oscillograms. (**d** and **g**) Average pupil diameters of the mice in dark (t_0_ ~ t_5_) and light (t_5_ ~ t_14_) conditions are measured and shown in graphs. **e** and **h** The values in the graphs are the contraction speed, which is defined as the time taken to reach the minimal pupil diameter from the maximum diameter (Δt = t_min_ – t_max_). Error bars are SD (*n* = 6 [*Vax1*^*+/+*^] and n = 7 [*Vax1*^*AA/AA*^]). *, *p* < 0.05; **, p < 0.01; ***, *p* < 0.005. **i** Head-fixed P45 *Vax1*^*+/+*^ and *Vax1*^*AA/AA*^ mice were positioned in a chamber surrounded by monitors, which displayed a gray background. The center positions of the pupils in the right and left eyes were marked and then tracked by the iSCAN rodent eye tracking system, while the mice exposed to the monitors displayed black and white vertical stripes (0.2 c/d), which were moving in the indicated directions for 30 seconds. The relative positions of the pupil centers of the right eyes against the position at t_0_ are plotted in the oscillograms. Peak positions of the pupil centers between 30 sec and 60 sec at horizontal (**j**) and vertical (**k**) axes were collected, and the averages are shown in graphs. Error bars are SDs (*n* = 6 [*Vax1*^*+/+*^] and n = 8 [*Vax1*^*AA/AA*^]).

Moreover, achiasmatic humans and dogs were also reported to have reduced visual acuity^42–44^. Interestingly, they commonly exhibited seesaw nystagmus—an out-of-phase vertical oscillation in the two eyes in the absence of a visual stimulus. We found that the eyes of P45 *Vax1*^*AA/AA*^ mice oscillated spontaneously in an oval track in the absence of visual stimuli (Fig. 8a, b; Supplementary video 17), whereas the eyes of *Vax1*^*+/+*^ littermate mice gazed stably (Fig. 8a, left column; Supplementary video 16). The oscillatory cycles of the two eyes of *Vax1*^*AA/AA*^ mice were out of phase in the vertical axis but in phase in the horizontal axis (Fig. 8a, right column; Supplementary Fig. 6), phenocopying the seesaw nystagmus of achiasmatic humans and dogs.

Spontaneous oscillations of *Vax1*^*AA/AA*^ mouse eyes were also evident during pupil contraction responses to binocular light illumination (Supplementary video 19). However, the efficiency and speed of pupil contraction were not significantly different between *Vax1*^*+/+*^ and *Vax1*^*AA/AA*^ mice (Fig. 8c–e; Supplementary video 18, 19). Interestingly, pupil contraction occurred first in the consensual (i.e., unstimulated) eyes and then in direct (i.e., stimulated) eyes of *Vax1*^*AA/AA*^ mice given monocular illumination (Fig. 8f–h; Supplementary video 21). This was in contrast to the patterns in *Vax1*^*+/+*^ mice, which exhibited immediate contraction of pupils in direct eyes, followed by contraction of the pupils of consensual eyes (Fig. 8f–h; Supplementary video 20). These results suggest that pupillary oculomotor outputs are sent in opposite routes in *Vax1*^*AA/AA*^ mice compared with *Vax1*^*+/+*^ mice.

### Vax1^AA/AA^ mice exhibit visuomotor anomalies

We also investigated the optokinetic reflex (OKR) of mouse eyes to moving objects^45^. *Vax1*^*+/+*^ mice rotate their eyes correspondingly and periodically in the direction of movement of black and white vertical stripes, which rotate clockwise or counter clockwise (Fig. 8i–k; Supplementary video 22 and 24); however, they did not rotate their eyes when the stripes converged, diverged, or stopped in the front eye field (Fig. 8i–k; Supplementary video 26, 28, and 30). Interestingly, the eyes of *Vax1*^*AA/AA*^ mice stopped moving when the stripes moved clockwise or counterclockwise (Fig. 8i–k; Supplementary video 23 and 25), whereas they exhibited see-saw nystagmus in response to other stimuli (Fig. 8i–k; Supplementary video 27, 29, and 31). These results suggest that *Vax1*^*AA/AA*^ mice might recognize the movement of objects; however, their visuomotor systems do not operate in the same way as they do in *Vax1*^*+/+*^ mice, which rotate their eyes and heads correspondingly to moving objects.

## Discussion

Many vertebrate organs exhibit bilateral symmetry. However, these paired organs are not simply duplications but instead are frequently functional complements of each other. For instance, the left hemisphere of the human cerebral cortex houses the language center, whereas the right hemisphere is where pattern recognition occurs^46^. This is demonstrated by the ‘split-brain’ phenomenon, in which an individual whose two cerebral hemispheres are not connected by the CC cannot match words to the corresponding objects. In vertebrate binocular visual systems, bilateral projection of RGC axons at the OC is necessary for the brain to receive visual information coming from the two eyes^4,5^. Therefore, proper development of the OC is required for overlapping focal-drifted images from each eye and coordinating bilateral oculomotor responses.

Agenesis of the OC (AOC) has been reported in various vertebrates, including humans, dogs, and fish^42–44,47^. However, the molecular features of AOC have not been identified except in *belladonna* (*bel*) zebrafishes, which carry mutations in the LIM homeobox 2 (*lhx2*) gene^48^. In achiasmatic *bel* mutants, ventral diencephalic regions, including the preoptic area (POA), vHT and OS, are not patterned properly due to the failure to express key genes, including *vax2*, *zic2.1*, and *pax2.1*^48^. However, Lhx2 and Pax2 are properly expressed in the *Vax1*^*AA/AA*^ mouse OS (Fig. 3c; Lhx2 data not shown). Vax2 and Zic2 are not present in the OS of *Vax1*^*+/+*^ and *Vax1*^*AA/AA*^ mice but are expressed in the ventral and ventral-temporal retina, respectively (Fig. 5c; Vax2 data not shown). Moreover, the OS was specified properly in *Vax1*^*AA/AA*^ mice, whereas the OS specification in *Vax1*^*−/−*^ mice was incomplete (Fig. 3c, d).

However, the OS in *Vax1*^*AA/AA*^ mice exhibited developmental delay. In these mice, the optic fissures at the OS were closed completely by E16.5, whereas in *Vax1*^*+/+*^ mice, they had already disappeared, and OS cells had spread among RGC axons in a salt-and-pepper pattern by E14.5 (Fig. 3c, d). The morphology of the E14.5 *Vax1*^*AA/AA*^ mouse OS was largely similar to that of E12.5 *Vax1*^*+/+*^ mice, whose OS cells exhibited neuroepithelial characteristics (Fig. 3b). The molecular mechanisms underlying the maturation of OS cells remain largely unknown, although several markers of OS cell lineage have been identified. For instance, Nestin and E-cad are expressed in the OS neuroepithelium; S100ß is expressed in OS APCs; and glial fibrillary acidic protein (Gfap) is expressed in OS astrocytes^49^. These markers, however, are not mutually exclusive. S100ß was detected together with E-cad in OS cells (Fig. 3d), suggesting an OS cell transition state between the neuroepithelium and APC. As a result, it is difficult to dissect the developmental stages of OS cells clearly with the limited information available. Thus, a more comprehensive understanding of OS maturation will require the identification of additional markers that are selectively expressed at specific OS developmental stages.

The patterning of the HT was also unlikely to be affected in *Vax1*^*AA/AA*^ mice (Fig. 5c), as had also been reported previously in *Vax1*^*−/−*^ mice^12,13^. However, the HT of E14.5 *Vax1*^*AA/AA*^ mouse embryos did not exhibit the normal structure seen in *Vax1*^*+/+*^ littermates. It protruded ventrally, whereas the vHT of *Vax1*^*+/+*^ mice was flattened over the RGC axons (Fig. 5a, b). The results suggest that the structural alteration of HT of *Vax1*^*AA/AA*^ mice could result from the absence of RGC axons, which might provide structural supports for vHT flattening. Alternatively, the OS, which formed a continuous neuroepithelial structure with the HT and failed to close the fissures (Fig. 3b), might also be related to the vHT phenotype of E14.5 *Vax1*^*AA/AA*^ mice. Finally, in addition to these extrinsic factors, structural alterations could be induced by intrinsic gene expression changes in vHT cells. However, the vHT patterning factor *Shh* was detected in a similar domain of *Vax1*^*+/+*^ and *Vax1*^*AA/AA*^ mouse HT (Fig. 5c). Glast-positive radial glial cells were also detected in the vHT of *Vax1*^*AA/AA*^ mice (Fig. 5c) and expressed Vegfa and Nr-Cam (Fig. 5d), which are known to support RGC axon growth toward the midline^9,10^. Therefore, the intrinsic factors that were changed in the vHT of *Vax1*^*AA/AA*^ mouse embryos to cause vHT malformation need to be identified by comprehensive analyses of vHT cells in future studies.

**Fig. 9:**
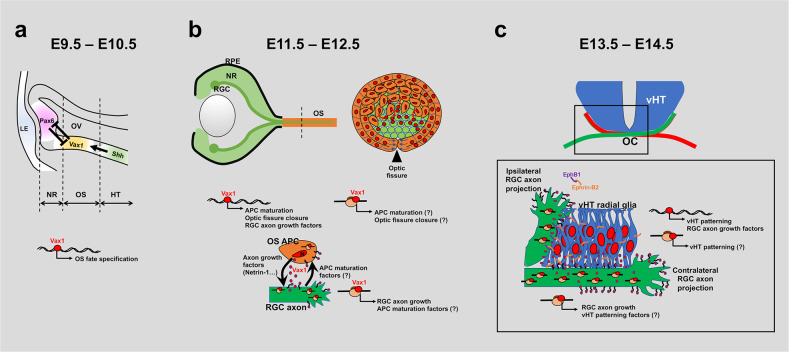
Schematic diagram depicting the pleiotropic roles of Vax1 in mouse optic nerve development. **a** Vax1 is expressed in the proximal optic vesicle (OV) downstream of the Shh signal, which comes from the ventral midline and specifies OS fate by suppressing the expression of retinal determinants, including Pax6^15^, between E9.5 and E10.5. **b** This event is followed by invagination of the OV that forms a double-layer optic cup of neural retina (NR) and RPE in the distal OV between E11.5 and E12.5. The fissures in the OS (arrowhead) and the optic cup are also closed as a result of invagination. Optic fissure closure was impaired in *Vax*^*−/−*^ mice but delayed in *Vax1*^*AA/AA*^ mice, which express secretion-defective and transcription-competent Vax1^AA^ mutants (Fig. 3b). These results suggest that Vax1 regulates fissure closure not only through the expression of target genes in the OS cell nucleus but also via secretion. The secreted Vax1 not only penetrates neighboring RGC axons but can also reenter OS APCs. Autocrine Vax1, in turn, might induce the expression of genes that regulate APC maturation and/or fissure closure, as OS APC-derived Vax1 in RGC axons promotes axonal growth via local mRNA translation^16^. APC maturation factors could also be included in the targets of Vax1 in RGC axons. Thus, APC maturation could also be delayed in *Vax1*^*AA/AA*^ mice, in which RGC axon growth is retarded. N, nucleus. **c** Vax1 could also act in neighboring RGC axons as well as vHT cells, as it did in the OS. Thus, defective secretion of Vax1 could not only affect the growth of RGC axons approaching the vHT but could also cause developmental disorders in the vHT. Therefore, bilateral projection of RGC axons is not only dependent on the expression of pathway selection cues, including Vegfa, Nr-Cam, and Ephrin-B2, in the vHT but also requires proper expression of vHT patterning genes.

It has been proposed that Vax1 specifies the OS in vertebrates by directly suppressing the expression of the retinal fate determinant Pax6^12,15^. Expression of Pax6 in the OS was suppressed properly in *Vax1*^*AA/AA*^ mice, whereas it was induced ectopically in the *Vax1*^*−/−*^ mouse OS (Fig. 3c). The ability of Vax1^AA^ to bind DNA sequences of the *Pax6 α*-enhancer was not different from that of Vax1 (Fig. 2e, f), suggesting that Vax1^AA^ could regulate target gene expression as efficiently as Vax1. These results suggest that Vax1 transcription factor activity is not crucial for OS maturation, whereas it is necessary for the specification of the OS. These results also suggest that secreted Vax1 supports OS maturation. The secreted Vax1 might function in autocrine and paracrine manners (Fig. 9b). The secreted Vax1 might reenter OS cells to induce target genes at the posttranscriptional level, as it did in neighboring RGC axons, to induce local mRNA translation of axon growth-stimulating genes^16^. In addition, OS-derived Vax1 in RGC axons might also induce the expression of the factors that trigger the maturation of OS cells. Therefore, Vax1 could play a role as a signaling factor that couples OS maturation and RGC axon growth. Previously, RGC-derived Shh was shown to promote OS cell proliferation and differentiation^50,51^, suggesting the possibility that OS-derived Vax1 induces *Shh* mRNA translation in axons. However, the deletion of *Shh* in the retina did not delay the closure of OS fissures^51^. Therefore, the delayed maturation of OS cells in *Vax1*^*AA/AA*^ mice might not result from Shh reduction in neighboring RGC axons.

Collectively, our study suggests pleiotropic roles of Vax1 in mouse optic nerve development. Vax1 is expressed in the mouse ventral forebrain after E8.5 and specifies the HT and OS in the ventral diencephalic area^12,13^. This might be mediated by transcriptional regulation of various target genes, including the negative target *Pax6*^15^ (Fig. 9a). In the OS, Vax1 not only induces the development of the OS cell lineage but also regulates the growth of neighboring RGC axons. The latter is not only promoted by Vax1-dependent expression of RGC axon growth factors, such as *Netrin-1* and *Sema5a* (Supplementary Fig. 3), in OS APCs but also requires intercellular transfer of the Vax1 protein, which enhances local mRNA translation in RGC axons^16^ (Fig. 9b). Therefore, not only the loss of Vax1 (i.e., knockout) but also the inactivation of Vax1 transfer (i.e., KA-to-AA mutation) could result in retarded RGC axon growth (Fig. 4b, c). These mutations also lead to the malformation of the vHT structure and defective RGC axon growth to the midline (Figs. 5a, 9c). Thus, the identification of transcription and translation targets of Vax1 in OS, vHT, and RGC axons should be considered in future studies to understand the pleiotropic roles of Vax1 in mouse optic nerve development.

One of the most prominent characteristics of achiasmatic animals is seesaw nystagmus^42–44^. In mammalian oculomotor systems, the rostral interstitial nucleus of the medial longitudinal fasciculus (riMLF) and the interstitial nucleus of Cajal (INC) in the tegmentum of the midbrain were identified as key regulatory centers for vertical gaze^52,53^. The riMLF neurons project directly to the oculomotor nucleus (ON) in the midbrain and indirectly via the INC to trigger ocular muscle contraction^52^. In *Vax1*^*+/+*^ mice, RGC axons bilaterally innervate brain areas (Fig. 6c–e), which send signals to riMLF neurons. Therefore, riMLF neurons in both hemispheres can be activated commonly even by monocular stimulation; consequently, the four ocular muscles—inferior rectus (IR), superior oblique (SO), inferior oblique (IO) and superior rectus (SR)—can contract simultaneously to result in vertical gaze. In contrast, the oculomotor centers in *Vax1*^*AA/AA*^ mice receive visual inputs only from the ipsilateral eyes (Fig. 6c). Therefore, signals from the right eye in *Vax1*^*AA/AA*^ mice might induce contractions of IR and IO in the right eye and SR and SO in the left eye, resulting in nasal-downward pendular motion of the right eye and temporal-upward pendular movement of the left eye (Fig. 8a; Supplementary Fig. 6; Supplementary video 17). This vertically out-of-phase but horizontally in-phase eye movement is then followed by temporal-upward and nasal-downward pendular motions, completing one oscillation cycle. This alternating eye movement, therefore, suggests that the signals from the two eyes are desynchronized and/or fed back on each other.

The paramedian pontine reticular formation (PPRF) is a horizontal gaze center in the pons that receives oculomotor inputs from the ipsilateral prefrontal cortex^53,54^. The PPRF also receives inputs from the contralateral SC and delivers them to the neighboring abducens nucleus (ABN). Abducens internuclear neurons (AIN) in the ABN then relay the signals to CN-III across the midline to stimulate the medial rectus (MR) ocular muscle, while abducens motor neurons (AMN) in the ABN connect ipsilaterally to the lateral rectus (LR). Given the binocular nature of efferent nerve fibers of ABN neurons, contractions of the MR in one eye and the LR in the other eye rotate both eyes in the same direction (Fig. 8i). Horizontal gaze is therefore achieved when MR and LR in the same eye are activated simultaneously. However, because of the seesaw nystagmus, horizontal eye gaze was not observed in *Vax1*^*AA/AA*^ mice.

The eyes of *Vax1*^*AA/AA*^ mice frequently stopped oscillating when mice were presented with objects moving clockwise or counterclockwise instead of rotating correspondingly in the direction of object movement as in *Vax1*^*+/+*^ mice (Fig. 8i; Supplementary videos 23 and 25). This stimulus-driven ectopic gaze of *Vax1*^*AA/AA*^ mouse eyes might result from the combinatorial activation of extraocular muscles. As noted above, the seesaw nystagmus of *Vax1*^*AA/AA*^ mice is likely driven by alternating activation of IR + IO and SR + SO ocular muscles. Therefore, IR + IO-driven nasal (and downward) movement of the right eye could be antagonized by LR-induced temporal movement in response to clockwise movement of the object. At the same time, SR + SO-driven temporal (and upward) movement of the left eye might be antagonized by MR-induced nasal movement. However, given the presence of the respective downward and upward forces after antagonism by the LR and MR, the right eye position is slightly below the center, and the left eye position is slightly above the center during the ectopic gaze in responding to clockwise stripe rotation (Fig. 8i).

The *bel rev* achiasmatic zebrafish model exhibits nystagmus when facing nonmoving stripes^55^. The nystagmus of the fish, however, disappears in the dark and after nonpatterned illumination of the visual field, which triggers seesaw nystagmus in achiasmatic mammals^42,56^ (Fig. 8a). Furthermore, in contrast to the stimulus-driven ectopic gaze in achiasmatic *Vax1*^*AA/AA*^ mice (Fig. 8i), *bel rev* zebrafish exhibit reversed horizontal OKR in response to the rotation of stripes^57,58^. It was proposed that the ipsilateral RGC projection supplies a reversed retinal slip velocity input to the optokinetic system in zebrafish to elicit eye movements that compensate for retinal slip in the wrong direction^57,58^. However, reversed OKR and postural abnormalities have not been reported in humans^42^, dogs^43,59^, or mice (Fig. 8i). These results suggest that the oculomotor circuits of zebrafish are likely different from those in mammals.

The oculomotor circuit also controls bilateral pupillary contraction, which is triggered by RGCs that are wired to the pretectal nucleus (PN) in the midbrain^60^. PN neurons relay these signals to the Edinger-Westphal nucleus (EWN), which projects axons to the ipsilateral CN-III to induce pupillary contraction^61,62^. It has been suggested that the EWN receives signals from ipsilateral and contralateral PNs to induce bilateral pupillary contraction. However, given the faster pupil contraction of directly stimulated eyes compared with consensual eyes in *Vax1*^*+/+*^ mice (Fig. 8f), the PN might primarily stimulate the contralateral EWN in mice. Consequently, the repeated midline crossings at the retina-PN and PN-EWN axes enable the directly stimulated eye to respond faster than the consensual eye. However, an ipsilateral retina-PN connection followed by a contralateral PN-EWN connection might result in an inverse order of pupillary contraction in *Vax1*^*AA/AA*^ mice. These results suggest that the operation of the oculomotor system depends on a constant number of midline crossings; therefore, the system cannot function properly if one of those commissures is missing.

The axons of SC neurons also project contralaterally to the cervical spinal cord through the tectospinal tract and can trigger head turns in response to visual stimuli^63^. Therefore, activation of the right SC, which receives a majority of its inputs from the left eye, which captures objects in the left visual field, predominantly contracts the left neck muscle to trigger a leftward head turn in *Vax1*^*+/+*^ mice. Given the exclusive ipsilateral retinocollicular connection, the spinal outputs in achiasmatic *Vax1*^*AA/AA*^ mice are likely inverse to those in *Vax1*^*+/+*^ mice. This might make *Vax1*^*AA/AA*^ mice turn their heads in the direction opposite the stimulus. *Vax1*^*AA/AA*^ mice, however, startled instead of turning their heads in the direction opposite the movement of horizontally drifting stripes (Fig. 7i; Supplementary video 14). These results suggest that head-turn behavior is not only determined by eye-SC-spinal cord circuits but, given the agenesis of the CC in *Vax1*^*AA/AA*^ mice (Fig. 6a), is also affected by cortical circuits that regulate the activity of the PPRF to pursue the objects^63^.

Our results show that retinogeniculate and retinocollicular connections are likely intact in *Vax1*^*AA/AA*^ mice (Fig. 6c, d), although they are wired exclusively in the ipsilateral paths. Furthermore, it might be possible that their visual perception is also preserved by reorganizing intracortical connections, as was proposed in achiasmatic human cases^64,65^. These results suggest that the impaired OKR of *Vax1*^*AA/AA*^ mice might result from defects in visuomotor responses triggered by ipsilaterally biased visual inputs. However, given the absence of CC (Fig. 6a) and reduced retinal activity (Fig. 7a), the anomalies can also be influenced by the split cerebral cortex and the retina with reduced cone photoreceptor activity.

## Supplementary information

Supplementary materials

Supplementary video 1

Supplementary video 2

Supplementary video 3

Supplementary video 4

Supplementary video 5

Supplementary video 6

Supplementary video 7

Supplementary video 8

Supplementary video 9

Supplementary video 10

Supplementary video 11

Supplementary video 12

Supplementary video 13

Supplementary video 14

Supplementary video 15

Supplementary video 16

Supplementary video 17

Supplementary video 18

Supplementary video 19

Supplementary video 20

Supplementary video 21

Supplementary video 22

Supplementary video 23

Supplementary video 24

Supplementary video 25

Supplementary video 26

Supplementary video 27

Supplementary video 28

Supplementary video 29

Supplementary video 30

Supplementary video 31

## References

[1] Seabrook TA, Burbridge TJ, Crair MC, Huberman AD (2017). Architecture, Function, and Assembly of the Mouse Visual System. Annu. Rev. Neurosci..

[2] Rusak B, Groos G (1982). Suprachiasmatic stimulation phase shifts rodent circadian rhythms. Science.

[3] Zhang C, Kolodkin AL, Wong RO, James RE (2017). Establishing Wiring Specificity in Visual System Circuits: From the Retina to the Brain. Annu. Rev. Neurosci..

[4] Petros TJ, Rebsam A, Mason CA (2008). Retinal axon growth at the optic chiasm: to cross or not to cross. Annu. Rev. Neurosci..

[5] Herrera E, Erskine L, Morenilla-Palao C (2019). Guidance of retinal axons in mammals. Semin. Cell. Dev. Biol..

[6] Rice DS, Williams RW, Goldowitz D (1995). Genetic control of retinal projections in inbred strains of albino mice. J. Comp. Neurol..

[7] Williams SE (2003). Ephrin-B2 and EphB1 mediate retinal axon divergence at the optic chiasm. Neuron.

[8] Peng J (2018). Sonic Hedgehog Is a Remotely Produced Cue that Controls Axon Guidance Trans-axonally at a Midline Choice Point. Neuron.

[9] Erskine L (2011). VEGF signaling through neuropilin 1 guides commissural axon crossing at the optic chiasm. Neuron.

[10] Williams SE (2006). A role for Nr-CAM in the patterning of binocular visual pathways. Neuron.

[11] Kuwajima T (2012). Optic chiasm presentation of Semaphorin6D in the context of Plexin-A1 and Nr-CAM promotes retinal axon midline crossing. Neuron.

[12] Bertuzzi S, Hindges R, Mui SH, O’Leary DD, Lemke G (1999). The homeodomain protein vax1 is required for axon guidance and major tract formation in the developing forebrain. Genes. Dev..

[13] Hallonet M, Hollemann T, Pieler T, Gruss P (1999). Vax1, a novel homeobox-containing gene, directs development of the basal forebrain and visual system. Genes. Dev..

[14] Slavotinek AM (2012). VAX1 mutation associated with microphthalmia, corpus callosum agenesis, and orofacial clefting: the first description of a VAX1 phenotype in humans. Hum. Mutat..

[15] Mui SH, Kim JW, Lemke G, Bertuzzi S (2005). Vax genes ventralize the embryonic eye. Genes. Dev..

[16] Kim, N. et al. Regulation of retinal axon growth by secreted Vax1 homeodomain protein. *eLife* **3**, e02671 (2014). 10.7554/eLife.02671 PMC4178304 25201875

[17] Marquardt T (2001). Pax6 is required for the multipotent state of retinal progenitor cells. Cell.

[18] Muzumdar MD, Tasic B, Miyamichi K, Li L, Luo L (2007). A global double-fluorescent Cre reporter mouse. Genesis.

[19] Wang Y (2019). Beta-catenin signaling regulates barrier-specific gene expression in circumventricular organ and ocular vasculatures. eLife.

[20] Park YG (2019). Protection of tissue physicochemical properties using polyfunctional crosslinkers. Nat. Biotechnol..

[21] Yilmaz M, Meister M (2013). Rapid innate defensive responses of mice to looming visual stimuli. Curr. Biol..

[22] Prusky GT, Alam NM, Beekman S, Douglas RM (2004). Rapid quantification of adult and developing mouse spatial vision using a virtual optomotor system. Invest. Ophthalmol. Vis. Sci..

[23] Beurdeley M (2012). Otx2 binding to perineuronal nets persistently regulates plasticity in the mature visual cortex. J. Neurosci..

[24] Miyata S, Komatsu Y, Yoshimura Y, Taya C, Kitagawa H (2012). Persistent cortical plasticity by upregulation of chondroitin 6-sulfation. Nat. Neurosci..

[25] Cardin AD, Weintraub HJ (1989). Molecular modeling of protein-glycosaminoglycan interactions. Arteriosclerosis.

[26] Vacik T, Stubbs JL, Lemke G (2011). A novel mechanism for the transcriptional regulation of Wnt signaling in development. Genes. Dev..

[27] Lee EJ (2019). Global Analysis of Intercellular Homeodomain Protein Transfer. Cell Rep..

[28] Deiner MS, Sretavan DW (1999). Altered midline axon pathways and ectopic neurons in the developing hypothalamus of netrin-1- and DCC-deficient mice. J. Neurosci..

[29] Oster SF, Bodeker MO, He F, Sretavan DW (2003). Invariant Sema5A inhibition serves an ensheathing function during optic nerve development. Development.

[30] Chiang C (1996). Cyclopia and defective axial patterning in mice lacking Sonic hedgehog gene function. Nature.

[31] Kim JW, Lemke G (2006). Hedgehog-regulated localization of Vax2 controls eye development. Genes. Dev..

[32] Herrera E (2003). Zic2 patterns binocular vision by specifying the uncrossed retinal projection. Cell.

[33] Soria JM (2004). Defective postnatal neurogenesis and disorganization of the rostral migratory stream in absence of the Vax1 homeobox gene. J. Neurosci..

[34] Bharti K, Gasper M, Bertuzzi S, Arnheiter H (2011). Lack of the ventral anterior homeodomain transcription factor VAX1 leads to induction of a second pituitary. Development.

[35] Luppi PH, Fort P, Jouvet M (1990). Iontophoretic application of unconjugated cholera toxin B subunit (CTb) combined with immunohistochemistry of neurochemical substances: a method for transmitter identification of retrogradely labeled neurons. Brain Res..

[36] Guido W (2018). Development, form, and function of the mouse visual thalamus. J. Neurophysiol..

[37] Huberman AD, Feller MB, Chapman B (2008). Mechanisms underlying development of visual maps and receptive fields. Annu. Rev. Neurosci..

[38] Lemke G, Reber M (2005). Retinotectal mapping: new insights from molecular genetics. Annu. Rev. Cell. Dev. Biol..

[39] Miura G, Wang MH, Ivers KM, Frishman LJ (2009). Retinal pathway origins of the pattern ERG of the mouse. Exp. Eye. Res..

[40] Chang B (2002). Retinal degeneration mutants in the mouse. Vis. Res..

[41] Sloane SA, Shea SL, Procter MM, Dewsbury DA (1978). Visual cliff performance in 10 species of muroid rodents. Anim. Learn. Behav..

[42] Apkarian P, Bour LJ, Barth PG, Wenniger-Prick L, Verbeeten B (1995). Non-decussating retinal-fugal fibre syndrome. An inborn achiasmatic malformation associated with visuotopic misrouting, visual evoked potential ipsilateral asymmetry and nystagmus. Brain.

[43] Dell’Osso LF, Williams RW (1995). Ocular motor abnormalities in achiasmatic mutant Belgian sheepdogs: unyoked eye movements in a mammal. Vis. Res.

[44] Williams RW, Hogan D, Garraghty PE (1994). Target recognition and visual maps in the thalamus of achiasmatic dogs. Nature.

[45] Cahill H, Nathans J (2008). The optokinetic reflex as a tool for quantitative analyses of nervous system function in mice: application to genetic and drug-induced variation. PLoS One.

[46] Wolman D (2012). The split brain: a tale of two halves. Nature.

[47] Karlstrom RO (1996). Zebrafish mutations affecting retinotectal axon pathfinding. Development.

[48] Seth A (2006). belladonna/(Ihx2) is required for neural patterning and midline axon guidance in the zebrafish forebrain. Development.

[49] Tao C, Zhang X (2014). Development of astrocytes in the vertebrate eye. Dev. Dyn..

[50] Burne JF, Raff MC (1997). Retinal ganglion cell axons drive the proliferation of astrocytes in the developing rodent optic nerve. Neuron.

[51] Dakubo GD (2003). Retinal ganglion cell-derived sonic hedgehog signaling is required for optic disc and stalk neuroepithelial cell development. Development.

[52] Büttner U, Büttner-Ennever JA, Rambold H, Helmchen C (2002). The contribution of midbrain circuits in the control of gaze. Ann. N. Y. Acad. Sci..

[53] Zee DS (1986). Brain stem and cerebellar deficits in eye movement control. Trans. Ophthalmol. Soc. U. K..

[54] Buttner-Ennever JA, Buttner U (1988). Neuroanatomy of the oculomotor system. The reticular formation. Rev. Oculomot. Res..

[55] Huang YY, Rinner O, Hedinger P, Liu SC, Neuhauss SC (2006). Oculomotor instabilities in zebrafish mutant belladonna: a behavioral model for congenital nystagmus caused by axonal misrouting. J. Neurosci..

[56] Dell’Osso LF, Williams RW, Jacobs JB, Erchul DM (1998). The congenital and see-saw nystagmus in the prototypical achiasma of canines: comparison to the human achiasmatic prototype. Vis. Res..

[57] Neuhauss SC (1999). Genetic disorders of vision revealed by a behavioral screen of 400 essential loci in zebrafish. J. Neurosci..

[58] Rick JM, Horschke I, Neuhauss SC (2000). Optokinetic behavior is reversed in achiasmatic mutant zebrafish larvae. Curr. Biol..

[59] Hogan D, Williams RW (1995). Analysis of the retinas and optic nerves of achiasmatic Belgian sheepdogs. J. Comp. Neurol..

[60] Szabadi E (2018). Functional Organization of the Sympathetic Pathways Controlling the Pupil: Light-Inhibited and Light-Stimulated Pathways. Front. Neurol..

[61] Hultborn H, Mori K, Tsukahara N (1978). The neuronal pathway subserving the pupillary light reflex. Brain Res..

[62] Kourouyan HD, Horton JC (1997). Transneuronal retinal input to the primate Edinger-Westphal nucleus. J. Comp. Neurol..

[63] Gandhi NJ, Katnani HA (2011). Motor functions of the superior colliculus. Annu. Rev. Neurosci..

[64] Hoffmann MB (2012). Plasticity and stability of the visual system in human achiasma. Neuron.

[65] Sinha P, Meng M (2012). Superimposed hemifields in primary visual cortex of achiasmic individuals. Neuron.

