# Supplementary material for: Visuomotor anomalies in achiasmatic mice expressing a transfer-defective Vax1 mutant
Source: Exp Mol Med. 2023 Feb 3;55(2):385–400. doi: 10.1038/s12276-023-00930-4 (PMC9981622; doi:10.1038/s12276-023-00930-4)
Supplement: Supplementary file 1 — Supplementary materials [file 12276_2023_930_MOESM1_ESM.docx]

**Supplementary Information**

**Visuomotor anomalies in achiasmatic mice expressing a transfer-defective Vax1 mutant**

Kwang Wook Min, Namsuk Kim, Jae Hoon Lee, Younghoon Sung, Museong Kim, Eun Jung Lee, Jong-Myeong Kim, Jae-Hyun Kim, Jaeyoung Lee, Wonjin Cho, Jee Myung Yang, Nury Kim, Jaehoon Kim, C. Justin Lee, Young-Gyun Park, Seung-Hee Lee, Han-Woong Lee, and Jin Woo Kim

Corresponding author :

Jin Woo Kim, Email: [jinwookim@kaist.ac.kr](mailto:jinwookim@kaist.ac.kr)

**This PDF file includes:**

Supplementary figures

Supplementary video information

Supplementary tables

**Supplementary Fig. 1. Generation of *Vax1^AA/AA^* mice.** **(a)** Structure of mouse *Vax1^AA^* gene (top) and its partial sequences of exon 2 that encodes AA replacing KR in the GAG binding motif (bottom). The substituted nucleotides for missense (red, KR to AA) and synonymous (green) mutations are shown. The amino acid sequences from the Vax1 and the Vax1^AA^ alleles are shown at the bottom of each nucleotide sequence. The relative positions of two crRNAs and ssODN are indicated in short blue bars and black bar with asterisk (*), respectively. **(b)** Top, relative positions of genotyping primers are indicated by arrows. The red asterisks (*) indicate KR to AA mutation. Bottom, PCR genotyping results using genotyping primers. **(c)** Ventral views of P0 mouse mouths (top row) and palates (bottom row). Arrows indicate the clefts of lip (top row) and palate (bottom row), respectively. **(d)** Ventral views of P30 mouse mouths (top row) and palates (bottom row). **(e)** Survival rates of *Vax1^AA/AA^* mice by the indicated ages in x-axis are provided. Numbers of mice examined were provided in the graph.

**Supplementary Fig. 2. Development of Brn3b-positive RGCs in *Vax1^+/+^* and *Vax1^AA/AA^* mouse retina.** (**a**) Distribution of RGCs in E14.5 mouse embryonic sections was determined by immunostaining of an RGC-specific marker, Brn3b. Nuclei of the cells in the sections were visualized by DAPI staining. (**b**) Relative numbers of Brn3b(+) RGCs in the sections are shown in the graph. The values are SD (n=8; 5 independent litters).

**Supplementary Fig. 3. Expression of *Netrin-1* and *Sema5A* in *Vax1^+/+^* and *Vax1^AA/AA^* mouse OS.** Expressions of *Netrin-1* (**a**) and *Sema5A* (**c**) mRNA in E14.5 mouse embryos with indicated genotypes were investigated by ISH. Images in the second and fourth rows are the magnified versions of boxed areas in the first and third rows. Intensities of *Netrin-1* (**b**) and *Sema5A* (**d**) ISH signals were quantified and relative values are shown in the graphs. The values are SD (n=4; 3 independent litters). *, p<0.05; **, p<0.01; n.s., not significant.

**Supplementary Fig. 4. The *ISH* signals of sense probes. (a)** Specificities of the *ISH* signals detecting *Shh* (Figure 5C), *Ephnb2* (Figure 5D), and *Vegfa* (Figure 5D) in the vHT of mouse embryonic sections were determined by the *ISH* with sense probes for the corresponding genes. **(b)** Specificities of the *ISH* signals detecting *Vax1* (Figure 2B), *Ephb1* (Figure 5E), and *Shh* (Figure 5E) in the mouse retinas were determined by the *ISH* with sense probes for the corresponding genes.

**Supplementary Fig. 5. Anatomical features of the retina and optic nerve in *Vax1^+/+^* and *Vax1^AA/AA^* mice.** **(a)** Lateral views of the eyes of P30 *Vax1^+/+^* and *Vax1^AA/AA^* littermate mice. **(b)** Longitudinal sections of optic nerves of P30 *Vax1^+/+^* and *Vax1^AA/AA^* littermate mice were stained by H&E and Oil Red-O to visualize the distribution of the cells and lipids in the nerves, respectively. The sections were also immunostained with the antibodies that recognize the indicated marker proteins. Pax2 and Gfap, astrocytes; CD31, endothelial cells; Olig2, oligodendrocytes. **(c)** Sections of the eyes of P30 *Vax1^+/+^* and *Vax1^AA/AA^* littermate mice were stained by H&E or the antibodies that recognize the indicated marker proteins. Rhodopsin, rod photoreceptors; M-opsin, M-cone photoreceptors; S-opsin, S-cone photoreceptors; Calbindin, horizontal cells and amacrine cell subset; Vsx2, bipolar cells; Pax6, amacrine cells; Brn3b, RGCs; Sox9, Müller glia; Gfap, astrocytes. **(d)** Relative numbers of Pax2-positive astrocytes and Olig2-positive oligodendrocytes are shown in a graph. **(e)** Relative numbers of retinal cells expressing the corresponding markers in *Vax1^AA/AA^* mice against *Vax1^+/+^* littermate mice are shown in the graph. Error bars denote SD. Numbers of samples (from independent litters) are provided in the graphs. *, *p*<0.05.

**Supplementary Fig. 6. Spontaneous movement of *Vax1^AA/AA^* mouse eyes.** Relative positions of the pupil centers in right and left eyes of head-fixed P45 *Vax1^+/+^* and *Vax1^AA/AA^* were recorded by the iSCAN rodent eye tracking system at every 8 msec for 10 secs while the mice were kept in dark. The positions were plotted in the graphs. The results show the eyes of *Vax1^AA/AA^* rotate spontaneously in an oval track while *Vax1^+/+^* mouse eyes keep their positions.

**Supplementary Videos information**

Supplementary video 1. Video presentation of 3D imaging data of RGC axons in E12.5 *Vax1^+/+^* mice.

Supplementary video 2. Video presentation of 3D imaging data of RGC axons in E12.5 *Vax1^AA/AA^* mice.

Supplementary video 3. Video presentation of 3D imaging data of RGC axons in E13.5 *Vax1^+/+^* mice.

Supplementary video 4. Video presentation of 3D imaging data of RGC axons in E13.5 *Vax1^AA/AA^* mice.

Supplementary video 5. Video presentation of 3D imaging data of RGC axons in E14.5 *Vax1^+/+^* mice.

Supplementary video 6. Video presentation of 3D imaging data of RGC axons in E14.5 *Vax1^AA/AA^* mice.

Supplementary video 7. Response of *Vax1^+/+^* mice to a looming shadow.

Supplementary video 8. Response of *Vax1^AA/AA^* mice to a looming shadow.

Supplementary video 9. Response of *Pde6b^rd1/rd1^* mice to a looming shadow.

Supplementary video 10. Cliff assay of *Vax1^+/+^* mice.

Supplementary video 11. Cliff assay of *Vax1^AA/AA^* mice.

Supplementary video 12. Cliff assay of *Pde6b^rd1/rd1^* mice.

Supplementary video 13. Optomotor response of *Vax1^+/+^* mice.

Supplementary video 14. Optomotor response of *Vax1^AA/AA^* mice.

Supplementary video 15. Optomotor response of *Pde6b^rd1/rd1^* mice.

Supplementary video 16. Eye movement of *Vax1^+/+^* mice in dark.

Supplementary video 17. Eye movement of *Vax1^AA/AA^* mice in dark.

Supplementary video 18. Pupil contraction of *Vax1^+/+^* mice after binocular light illumination.

Supplementary video 19. Pupil contraction of *Vax1^AA/AA^* mice after binocular light illumination.

Supplementary video 20. Pupil contraction of *Vax1^+/+^* mice after monocular light illumination.

Supplementary video 21. Pupil contraction of *Vax1^AA/AA^* mice after monocular light illumination.

Supplementary video 22. Eye movement of *Vax1^+/+^* mice in response to vertical stripes rotating clockwise direction.

Supplementary video 23. Eye movement of *Vax1^AA/AA^* mice in response to vertical stripes rotating clockwise direction.

Supplementary video 24. Eye movement of *Vax1^+/+^* mice in response to vertical stripes rotating counter clockwise direction.

Supplementary video 25. Eye movement of *Vax1^AA/AA^* mice in response to vertical stripes rotating counter clockwise direction.

Supplementary video 26. Eye movement of *Vax1^+/+^* mice in response to converging vertical stripes.

Supplementary video 27. Eye movement of *Vax1^AA/AA^* mice in response to converging vertical stripes.

Supplementary video 28. Eye movement of *Vax1^+/+^* mice in response to diverging vertical stripes.

Supplementary video 29. Eye movement of *Vax1^AA/AA^* mice in response to diverging vertical stripes.

Supplementary video 30. Eye movement of *Vax1^+/+^* mice in response to stationary vertical stripes.

Supplementary video 31. Eye movement of *Vax1^AA/AA^* mice in response to stationary vertical stripes.

| **Supplementary table 1. Information of the reagents and resources.** | | | | |
| --- | --- | --- | --- | --- |
| **Reagent type (species) or resource** | **Designation** | **Source or reference** | **Identifiers** | **Additional information** |
| Antibody | Anti-ß-actin (Rabbit polyclonal) | Santa cruz biotechnology | SC-1616 | 1:1000 |
| Antibody | Anti-Brn3b (Goat polyclonal) | Santa cruz biotechnology | SC-31989 | 1:200 |
| Antibody | Anti-CD31 (Hamster polyclonal) | Millipore | MAB1398Z | 1:200 |
| Antibody | Anti-E-cadherin (Mouse monoclonal) | BD | 610181 | 1:200 |
| Antibody | Anti-GFAP (Rabbit polyclonal) | Abcam | AB48050 | 1:200 |
| Antibody | Anti-GFP (Rabbit polyclonal) | Abcam | AB290 | 1:200 |
| Antibody | Anti-GFP (Mouse monoclonal) | Santa cruz biotechnology | SC-9996 | 1:200 |
| Antibody | Anti-GLAST (Guinia pig polyclonal) | Millipore | AB1782 | 1:500 |
| Antibody | Anti-L1-CAM (Rat monoclonal) | Millipore | MAB5272 | 1:200 |
| Antibody | Anti-M-opsin (Mouse monoclonal) | Millipore | AB5405 | 1:200 |
| Antibody | Anti-Nestin (Mouse monoclonal) | Millipore | MAB353 | 1:100 |
| Antibody | Anti-NF160 (Mouse monoclonal) | DSHB | 2H3 | 1:50 |
| Antibody | Anti-Nr-CAM (Rabbit polyclonal) | Abcam | AB24344 | 1:200 |
| Antibody | Anti-Olig2 (Rabbit polyclonal) | Millipore | AB9610 | 1:200 |
| Antibody | Anti-Pax2 (Rabbit polyclonal) | Invitrogen | 71-6000 | 1:200 |
| Antibody | Anti-Pax6 (Rabbit polyclonal) | Covance | PRB-278P | 1:200 |
| Antibody | Anti-Pax6 (Mouse monoclonal) | Santa cruz biotechnology | SC-81649 | 1:200 |
| Antibody | Anti-Rhodopsin (Mouse monoclonal) | Chemicon | MAB5356 | 1:200 |
| Antibody | Anti-S-opsin (Rabbit polyclonal) | Millipore | AB5407 | 1:200 |
| Antibody | Anti-S100ß (Rabbit polyclonal) | Abcam | AB52642 | 1:500 |
| Antibody | Anti-Sox9 (Rabbit polyclonal) | Santa cruz biotechnology | SC-20095 | 1:200 |
| Antibody | Anti-Vax1 (Rabbit polyclonal) | Mui et al. (2005) | N.A. | 1:50 |
| Antibody | Anti-Vsx2 (Guinia pig polyclonal) | gift from Dr. Mi-Ryoung Song (GIST) | N.A. | 1:200 |
| Antibody | Anti-Zic2 (Rabbit polyclonal) | Millipore | AB15392 | 1:200 |
| Mouse | Vax1^AA/AA^ | In this study |  |  |
| Mouse | Vax1^tm1Grl^ | Bertuzzi et al. (1999) |  |  |
| Mouse | Pax6 α-Cre | Marquardt et al. (2001) |  |  |
| Mouse | *Gt(ROSA)26Sor^tm4(ACTB-tdTomato,-EGFP)Luo^/J* | Jackson Laboratory | 007676 |  |
| Mouse | *Gt(ROSA)26Sor^tm11(CAG-tdTomato*,-GFP*)Nat^/J* | Jackson Laboratory | 030867 |  |
| Cell line (Homo-sapiens) | Human cervical cancer HeLa cell | ATCC | PRID:CVCL_0030 |  |
| Cell line (Homo-sapiens) | Human embryonic kidney 293T | ATCC | PRID:CVCL_0063 |  |
| DNA construct | pCAGIG-V5-IRES-EGFP | In this study | N/A |  |
| DNA construct | pXBGFP-SDC2 | In this study | N/A |  |
| Chemical | SHIELD epoxy resin | CVC thermoset | GE-38 | SHIELD solution |
| Chemical | Paraformaldehyde | Sigma-Aldrich | P6148 | SHIELD solution |
| Chemical | Phosphate buffer | Sigma-Aldrich | P3619 | SHIELD solution |
| Chemical | Sodium carbonate | Sigma-Aldrich | S7795 | SHIELD solution |
| Chemical | Sodium bicarbonate | Sigma-Aldrich | S5761 | SHIELD solution |
| Chemical | Sodium dodecyl sulfate (SDS) | Sigma-Aldrich | L3771 | SDS clearing solution |
| Chemical | Sodium sulfite | Sigma-Aldrich | S0505 | SDS clearing solution |
| Chemical | Boric acid | Sigma-Aldrich | B6768 | SDS clearing solution |
| Chemical | Sodium hydroxide | Sigma-Aldrich | S5881 | SDS clearing solution |
| Chemical | Agarose | Sigma-Aldrich | A6013 | SDS clearing solution |
| Chemical | Dimethyl sulfoxide (DMSO) | Sigma-Aldrich | 0231 | Optical clearing solution |
| Chemical | 2,2-Thiodiethanol | Sigma-Aldrich | 88561 | Optical clearing solution |
| Chemical | Iohexol | Sigma-Aldrich | D2158 | Optical clearing solution |
| Chemical | Polyethylenimine (PEI) | Polysciens | 23966 |  |
| Chemical | SuperSignal West Pico plus chemiluminescent substratwe | Thermo Scientific | 34580 |  |
| Chemical | Heparin | Millipore | M535142 |  |
| Chemical | Protease inhibitor | Millipore | M535142 |  |
| Chemical | Hoechst 33342 | Invitrogen | H1399 |  |
| Chemical | 2,2,2-tribromoethanol | Sigma-Aldrich | T48402 |  |
| Software | Fluoview 4.0 | Olympus corporation | N/A | https://www.olympus-lifescience.com |
| Software | Imaris 9.3 | Bitplane | N/A | https://www.imaris.oxinst.com |
| Software | ZEN | Zeiss | N/A | https://www.zeiss.com |
| Software | GraphPad Prism v7.0 | GraphPad software | N/A | https://www.graphpad.com |
| Software | Ethovision XT10 | Noldus | N/A | https://www.imaris.oxinst.com |
| Software | Optomotry | Cerebral Mechanics | N/A | http://cerebralmechanics.com/ |
| Software | ISCAN | ISCAN Inc. | N/A | https://iscaninc.com |
| Software | ERG | Phoenix Research Labs | N/A | https://phoenixmicron.com |

| **Supplementary table 2. Information of oligonucleotides.** | | |
| --- | --- | --- |
| Oligonucleotides | Source | Identifier |
| Primer: *Vax1^AA^* WT-Forward:  AAG AGG ACT CGC ACG TCC | In this study | N/A |
| Primer: *Vax1^AA^* GT-Reverse:  AGG AAA GGC AAG CTG TCA TC | In this study | N/A |
| Primer: *Vax1^AA^* GT-Forward:  AGT ATG AAG TTA GCC CCC TTG G | In this study | N/A |
| Primer: *Vax1^AA^* KI-Reverse:  GTA AAC GAG GTC CTG GTC GC | In this study | N/A |
| Primer: Vax1^tm1Grl^ Forward:  AAT CAA TTG CAA CAG CGA G | Bertuzzi et al. (1999) | N/A |
| Primer: Vax1^tm1Grl^ Reverse:  AGA AGG AGG GTG GGA AAA GAA G | Bertuzzi et al. (1999) | N/A |
| Primer: Vax1^tm1Grl^ Mutant reverse:  ACC ACA GAT GAA ACG CCG AG | Bertuzzi et al. (1999) | N/A |
